# *GSLR* Drives Lung Adenocarcinoma as a Critical Regulator of Energy Homeostasis Through Creatine Kinase B Activation

**DOI:** 10.1002/advs.77950

**Published:** 2026-09-27

**Authors:** Xinyi Qian, Juze Yang, Jiayi Ren, Xiaowei Mao, Jia Li, Tengfei Sun, Liangliang Dong, Enguo Chen, Yan Lu, Pengyuan Liu

**Affiliations:** ^1^ Department of Pulmonary and Critical Care Medicine Regional Medical Center For National Institute of Respiratory Diseases Sir Run Run Shaw Hospital and Institute of Translational Medicine Zhejiang University School of Medicine Hangzhou Zhejiang China; ^2^ Zhejiang Key Laboratory of Precision Diagnosis and Therapy for Major Gynecological Diseases Department of Gynecologic Oncology Women's Hospital and Institute of Translational Medicine Zhejiang University School of Medicine Hangzhou Zhejiang China; ^3^ Zhejiang Key Laboratory of Frontier Medical Research on Cancer Metabolism Zhejiang University School of Medicine Hangzhou Zhejiang China

**Keywords:** energy homeostasis, epigenetic regulation, long non‐coding RNA, lung adenocarcinoma

## Abstract

Lung cancer remains the leading cause of cancer‐related mortality, with extremely high energy demands during progression. Long non‐coding RNAs (lncRNAs) have emerged as crucial regulators in cancer metabolism; however, their role in reprogramming energy metabolism in lung cancer remains incompletely understood. In this study, we identify a glucose/glutamine sensitive lncRNA, *GSLR*, as a driver of lung adenocarcinoma (LUAD) progression. *GSLR* is markedly upregulated in LUAD, and its high expression is associated with poor patient prognosis. Nutrient restriction reduces chromatin accessibility at the *GSLR* locus via altered histone marks, whereas CTCF activates its transcription. Mechanistically, *GSLR* directly binds the RNA helicase DHX9 and recruits it to the creatine kinase B (CKB) promoter, arresting R‐loop accumulation and thereby promoting CKB transcription. Elevated CKB expression sustains intracellular ATP homeostasis, thereby stabilizing mitochondrial membrane potential, preventing calcium overload, and reducing reactive oxygen species (ROS) accumulation. Collectively, our findings identify *GSLR* as a novel regulator of energy homeostasis that promotes lung cancer progression through the *GSLR*/DHX9/CKB axis. Targeting *GSLR* may thus represent a promising therapeutic strategy for LUAD.

## Graphical abstract

This study identifies the glucose/glutamine sensitive lncRNA GSLR as a driver of lung adenocarcinoma progression. Nutrient restriction reduces chromatin accessibility at the GSLR locus and blocks CTCF‐mediated transcriptional regulation. GSLR directly interacts with DHX9 and recruits it to the CKB promoter, arresting R‐loop accumulation and promoting CKB transcription. Elevated CKB sustains intracellular energy homeostasis, preserves mitochondrial integrity and suppresses apoptosis.

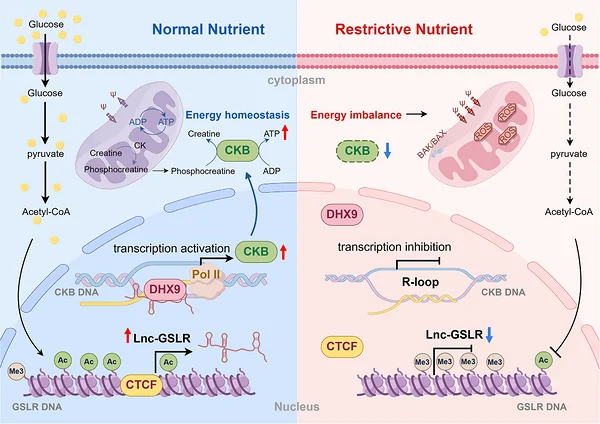

**Abbreviations**
CCK‐8: Cell Counting Kit 8
ChIP: Chromatin Immunoprecipitation
CKB: Creatine Kinase B
CTCF: CCCTC‐Binding Factor
DHX9: DExH‐Box Helicase 9
DRIP: DNA‐RNA Immunoprecipitation
ECAR: Extracellular Acidification Rate
EdU: Ethynyl Deoxyuridine
FISH: Fluorescence in Situ Hybridization
*GSLR*
: Glucose/Glutamine Sensitive lncRNA
LncRNA: Long Non‐coding RNA
LUAD: Lung Adenocarcinoma
ΔΨm: Mitochondrial Membrane Potential
mPTP: Mitochondrial permeability transition pore
NSCLC: Non‐small Cell Lung Cancer
OCR: Oxygen Consumption Rate
RIP: RNA Immunoprecipitation
ROS: Reactive Oxygen Species

## 1 Introduction

Lung cancer remains the leading cause of cancer‐related mortality worldwide, accounting for approximately 1.8 million deaths annually [1, 2]. Lung adenocarcinoma (LUAD) comprises about 40% of cases and has poor outcomes, with 5‐year survival rates of approximately 15% in advanced disease [3, 4]. Metabolic reprogramming has been established as a fundamental hallmark of cancer [5], with LUAD exhibiting pronounced alterations in multiple metabolic pathways [6, 7, 8]. In particular, the dysregulated utilization of glucose and glutamine, key fuels for proliferation, has emerged as a promising therapeutic vulnerability [9]. Growing evidence suggests that targeted modulation of these nutrient‐dependent pathways may open new avenues for cancer therapy [10, 11].

Recent studies have revealed that nutrient restriction can effectively suppress tumor growth through diverse mechanisms. For instance, Wu et al. demonstrated that reduced levels of the glycolytic intermediate 3‐phosphoglycerate (3‐PGA) alter the activity of phosphoglycerate dehydrogenase (PHGDH). Under nutrient limitation, PHGDH abandons its canonical role in serine biosynthesis and instead facilitates p53‐mediated apoptosis [12]. A separate study showed that glucose restriction in SLC7A11‐high cancer cells causes dramatic intracellular cystine accumulation. This leads to collapse of the cellular redox system and rapid cell death [13]. These findings collectively underscore the therapeutic potential of exploiting metabolic dependencies in cancer cells.

Long non‐coding RNAs (lncRNAs), defined as transcripts exceeding 200 nucleotides in length with limited protein‐coding capacity, have emerged as critical regulators of cellular physiology and pathology [14, 15, 16]. In cancer metabolism, lncRNAs mediate adaptive responses to metabolic stress and orchestrate reprogramming events that support tumor progression [17, 18, 19, 20]. Mounting evidence demonstrates that lncRNAs function as molecular sensors and effectors, enabling cancer cells to navigate metabolic challenges and sustain their proliferative demands [21, 22]. For example, the energy stress‐responsive lncRNA GAS5 modulates mitochondrial metabolism by regulating MDH2 acetylation and promoting formation of the FH‐MDH2‐CS metabolic compartment [23]. Similarly, high glucose‐induced LINC00842 binds to acetylated PGC‐1α and prevents its deacetylation by SIRT1. As a result, cellular metabolism shifts from mitochondrial oxidative catabolism to fatty acid synthesis, which drives the aggressive phenotype of pancreatic ductal adenocarcinoma (PDAC) [24]. These findings highlight a critical role for lncRNAs in linking environmental nutrient signals to metabolic regulation, ultimately promoting cancer progression.

In the present study, we identified a glucose/glutamine sensitive lncRNA (*GSLR*) that is markedly upregulated in lung adenocarcinoma (LUAD) tissues and positively correlated with poor prognosis. Epigenetically, nutrient restriction reduces chromatin accessibility at the *GSLR* locus by altering histone modifications, while the transcription factor CTCF activates its expression. Functionally, *GSLR* promotes LUAD cell proliferation and metastasis. Mechanistically, *GSLR* binds the RNA helicase DHX9 and recruits it to the creatine kinase B (CKB) promoter to enhance CKB transcription. The resulting *GSLR*/DHX9/CKB axis maintains energetic homeostasis by stabilizing mitochondrial membrane potential, preventing Ca^2^^+^ overload, and limiting reactive oxygen species, thereby modulating apoptosis. These findings position *GSLR* as a lncRNA mediator of metabolic adaptation in LUAD and a potential target for therapeutic intervention.

## 2 Methods

### 2.1 Cell Culture

Human LUAD cell lines A549 and H1650 and human embryonic kidney 293T (HEK‐293T) were purchased from the American Type Culture Collection (ATCC). A549 and H1650 cell lines were cultured in RPMI 1640 Medium. HEK‐293T cell line was cultured in DMEM. All medium was supplemented with 10% fetal bovine serum and 1% penicillin/streptomycin. All cell lines were cultured in a humidified atmosphere with 5% CO_2_ and at 37°C.

### 2.2 Plasmid Construction and Transfection

Specific shRNA sequences targeting the indicated genes (Table S1) were designed and constructed into pLKO.1 plasmid (restriction sites AgeI and EcoRI). The transfer plasmid (pLKO.1‐shRNA), packaging plasmid (psPAX2) and envelope plasmid (pCMV‐VSVG) were transfected into HEK‐293T cells using Lipofectamine 3000 transfection reagent (Invitrogen). After 48 h, the lentivirus suspension was collected, filtered through a 0.22 µm filter, and infected with target cells with 8 µg/ml polybrene. The stable knockdown cell lines were obtained by screening with 5 µg/ml puromycin for about one week.

Specific siRNA sequences targeting the indicated genes (Table S1) were designed and synthesized by GenePharma (Shanghai). Cells were transfected using GeneMute transfection reagent with siRNA at a final concentration of 50 nM. The knockdown efficiency of the target gene was assessed 48 h after transfection.

For overexpression, the full‐length sequence of GSLR (ENST00000536835.3) was amplified by PCR from cDNA and constructed into pcDNA3.1 plasmid (restriction sites EcoR I and Xho I). The plasmids were transfected into target cells using lipofectamine 3000 transfection reagent. The overexpression efficiency of the target gene was assessed 48 h after transfection.

All shRNA sequences, siRNA sequences, and PCR primers used in this study are listed in Table S1.

### 2.3 RNA Isolation and RT‐qPCR

Total RNA from cells and tissues was extracted using Trizol reagent (Invitrogen). 1 µg of total RNA was reverse transcribed into cDNA using the HiScript II Reverse Transcriptase SuperMix (Vazyme, #R233). Quantitative PCR was performed with ChamQ Universal SYBR qPCR Master Mix (Vazyme, #Q711). β‐actin and GAPDH served as endogenous controls. Relative gene expression was calculated using the 2^−△△Ct^ method. Primer sequences are listed in Table S1.

### 2.4 Fluorescence In Situ Hybridization (FISH) Assays

Custom‐designed Cy3‐labeled probes targeting *GSLR* were synthesized by RiBoBio (Guangzhou, China), with U6 snRNA and 18S rRNA probes serving as nuclear and cytoplasmic reference controls respectively. Cells were seeded onto sterile poly‐L‐lysine‐coated chamber slides at 60–70% confluence and allowed to adhere overnight. Cells were then fixed with 4% paraformaldehyde and permeabilized with 0.5% Triton X‐100. After three washes with PBS, cells were blocked with prehybridization solution at 37°C for 30 min. Hybridization was performed overnight at 37°C with probe hybridization solution containing Cy3‐labeled *GSLR* probes or reference probes in the dark. After the hybridization, cells were washed with hybridization wash buffer at 42°C to reduce background signal. Nuclei were counterstained with DAPI for 10 min before mounting with antifade medium. Fluorescence images were acquired using a Nikon A1 Ti confocal microscope.

### 2.5 Cell Proliferation Assays

For CCK‐8 assays, 1000 cells per well were seeded on 96‐well plates. After adherence, Cell Counting Kit‐8 (MCE, #HY‐K0301) was added at a ratio of 100:1 (medium:reagent) and incubated at 37°C for 2 h. The OD values were measured by SpectraMax M5 (Molecular Devices) at a wavelength of 450 nm.

For colony formation assays, 500 cells per well were plated in 12‐well plates and cultured for 7 days. Colonies were fixed with methanol and stained with 0.1% crystal violet.

### 2.6 Cell Migration Assays

Cell migration was assessed using trans‐well chambers (Millipore, #MCEP24H48). Briefly, a total of 30,000 cells were seeded in the upper of chamber using 0.3 mL of medium without serum, while 0.5 mL complete medium was introduced into the bottom of the chamber. After 12–24 h, migrated cells on the lower surface of the membrane were fixed with methanol and stained with 0.1% crystal violet. Images were taken by Leica DM4000 microscope.

### 2.7 Ethynyl Deoxyuridine (EdU) Incorporation Assays

DNA synthesis was evaluated using the EdU Apollo 488 DNA In Vitro Kit (RIBOBIO, #C10310‐3) according to the manufacturer's protocol. Briefly, 5000 cells per well were seeded into 96‐well plates and allowed to adhere overnight. The culture medium was then replaced with 100 µL of fresh medium containing 50 µM EdU, followed by incubation for 2 h at 37°C in a 5% CO_2_ humidified incubator. After labeling, cells were fixed with 4% paraformaldehyde for 15 min at room temperature, and the reaction was quenched with glycine for 5 min. Following permeabilization with 0.5% Triton X‐100 for 10 min, cells were stained with 100 µL of 1 × Apollo 488 reaction solution (protected from light) for 30 min at room temperature. Nuclei were counterstained with 1 × Hoechst 33342 for 10 min. Fluorescent images were acquired using a Zeiss Axio Observer A1 fluorescence microscope.

### 2.8 Extracellular Flux Analysis

Extracellular acidification rates (ECAR) and oxygen consumption rates (OCR) were analyzed by Seahorse Flux Analyzer XF96 (Agilent) according to the manufacturer's instructions. For assay preparation, cells were seeded into the Seahorse XF Microplate at a predetermined density and cultured overnight at 37°C in a 5% CO_2_ cell incubator. The sensor cartridge was hydrated in Seahorse XF Calibrant at 37°C in a non‐CO_2_ incubator overnight.

For glycolytic function assessment (ECAR measurements), cells were maintained in prewarmed XF Base Medium supplemented with 2 mM glutamine. After baseline measurements, sequential injections were performed as 10 mM glucose (to assess glycolysis), 1 µM oligomycin (to measure glycolytic capacity), and 50 mM 2‐deoxyglucose (to determine non‐glycolytic acidification).

Mitochondrial respiration (OCR) was evaluated using XF Base Medium containing 1 mM sodium pyruvate, 2 mM glutamine, and 10 mM glucose. Mitochondrial stress test compounds were injected as 1 µM oligomycin (ATP‐linked respiration), 1 µM FCCP (maximal respiration), and 0.5 µM rotenone/antimycin A (non‐mitochondrial respiration). All assay solutions were pH‐adjusted to 7.4 and prewarmed to 37°C prior to use.

### 2.9 Western Blotting

RIPA buffer (Beyotime, #P0013B) supplemented with 1 mM PMSF (Beyotime, #ST506) was utilized to lyse cells for 30 min on ice. The lysate was quantified using a BCA protein assay kit (Thermo Scientific, #23227) and subjected to denaturation at a temperature of 100°C for 10 min in the mixture of loading buffer (Fdbio, #FD002). Next, denatured proteins were separated using an SDS‐PAGE gel and subsequently transferred onto a 0.45 µm PVDF membrane (Merck Millipore, #IPVH00010). After blocking with 5% skim milk at room temperature for 2 h, the membrane was incubated with specific primary antibody at 4°C overnight. Afterward, secondary antibody conjugated with HRP incubated the membrane at room temperature for 1 h. Detection of protein signals was performed using ECL luminescence reagent (Meilunbio, #MA0186‐2). Images were taken with Bio‐Rad ChemiDoc Touch. All primary and secondary antibodies used in this study are listed in Table S2.

### 2.10 RNA Pull Down Assays

Biotin‐labeled lncRNA was transcribed in vitro using RNAmax‐T7 biotin‐labeled transcription kit (Ribio, C11002‐1). RNA pull down assays were performed using Pierce Magnetic RNA‐Protein Pull‐Down Kit (Thermo Fisher Scientific, #20164) according to the manufacturer's instructions. In brief, 25 pmol of biotin‐labeled RNA was incubated with 50 µl of streptavidin magnetic beads at room temperature for 1 h with agitation. Subsequently, cell lysates were introduced into RNA‐bound beads incubated at 4°C overnight with rotation. RNA‐Binding Protein Complexes underwent three washes using wash buffer and were subsequently eluted using elution buffer at a temperature of 37°C for 30 min with agitation. The eluted samples were denatured by 1 × SDS loading buffer and applied to downstream analysis.

### 2.11 RNA Immunoprecipitation (RIP)

RIP assays were performed using the Magna RIP RNA‐Binding Protein Immunoprecipitation Kit (Millipore, #17‐701). Briefly, approximately 2 × 10^7^ cells were harvested and lysed with RIP Lysis buffer. For each reaction, 5 µg of specific antibody (detailed information in Table S2) was incubated with 50 µl protein A/G magnetic beads at room temperature for 1 h with gentle rotation. At the end of the incubation, the beads were washed three times with washing buffer. The antibody‐beads complexes were incubated with cell lysates overnight at 4°C and washed five times with wash buffer at the end of the incubation. RNA‐protein complexes were eluted by treatment with Proteinase K for 30 min at 55°C, followed by RNA extraction using phenol: chloroform: isoamyl alcohol (125:24:1). The purified RNA was dissolved in 20 µl RNase‐free water and analyzed by RT‐qPCR. Input controls (1% of total lysate) and negative controls (normal IgG) were processed in parallel.

### 2.12 Chromatin Immunoprecipitation (ChIP)

ChIP assays were performed using the SimpleChIP Enzymatic Chromatin IP Kit (Magnetic Beads) (Cell Signaling Technology, #9003) following the manufacturer's protocol. Briefly, approximately 4 × 10^6^ cells were crosslinked with 1% formaldehyde (Sigma‐Aldrich) for 10 min at room temperature, followed by quenching with glycine for 5 min. Cells were then lysed and chromatin was digested to 150–900 bp fragments using micrococcal nuclease (37°C for 20 min) combined with sonication (for 4 min alternating 20s on and 20s off). For each reaction, 10–20 µg of digested, cross‐linked chromatin was incubated overnight at 4°C with specific antibodies (detailed information in Table S2), followed by capture with Pierce Protein G Magnetic Beads for 2 h at 4°C. After extensive washing, ChIP DNA was eluted and purified using spin columns, then quantified by qPCR. Normal IgG and histone H3 antibody served as negative and positive controls, respectively.

### 2.13 DNA‐RNA Immunoprecipitation (DRIP)

DRIP assays were performed as previously described [25] with minor modifications. Briefly, total nucleic acids were extracted from A549 cells by SDS/proteinase K treatment at 37°C overnight, followed by phenol/chloroform extraction and ethanol precipitation. DNA was fragmented using restriction enzyme cocktail (HindIII, EcoRI, BsrGI, XbaI and SspI) overnight. After phenol/chloroform extraction and ethanol precipitation, 8 µg DNA from each sample was subjected to immunoprecipitation with 10 µg of S9.6 antibody (Millipore, #MABE1095). Immunoprecipitated DNA was analyzed by qPCR using primers listed in Table S1.

### 2.14 ATP Assays

Cellular ATP levels were quantified using an ATP assay kit (Beyotime, #S0027). Briefly, cells cultured in 6‐well plates were lysed with 200 µL of ice‐cold lysis buffer and incubated on ice for 10 min. A standard curve was prepared using ATP standards at concentrations of 0.01, 0.1, 0.3, 1, 3, and 10 µM in lysis buffer. For measurement, 100 µl of ATP detection working solution was added to a 96‐well white opaque plate and incubated at room temperature for 5 min. Subsequently, 20 µl of sample or standard was added to each well and mixed immediately by gentle pipetting. Luminescence was measured within 2 s using a SpectraMax M5 microplate reader (Molecular Devices). ATP concentrations were calculated by comparing the relative light units (RLU) to the standard curve. All measurements were performed in triplicate, and values were normalized to total protein concentration determined by BCA assay.

### 2.15 Reactive Oxygen Species (ROS) Assays

Intracellular ROS levels were detected using the Reactive Oxygen Species Assay Kit (Beyotime, #S0033). Briefly, the fluorescent probe DCFH‐DA was diluted in serum‐free medium at 1:1000 to a final concentration of 10 µM. Cells grown in 6‐well plates were harvested and resuspended in DCFH‐DA working solution. After incubation at 37°C in a 5% CO_2_ humidified incubator for 20 min with gentle inversion every 3–5 min to ensure uniform labeling, cells were washed three times with warm serum‐free medium to completely remove extracellular probe. The fluorescence intensity (excitation/emission: 488/525 nm) was measured within 30 min using a CytoFLEX S flow cytometer (Beckman).

### 2.16 Mitochondrial Membrane Potential Measurements

Cells were seeded in confocal dishes at an appropriate density and allowed to adhere overnight in complete medium at 37°C with 5% CO_2_. The JC‐1 working solution (4 µM in serum‐free medium) was then added and cells were incubated for 30 min under standard culture conditions. Following incubation, cells were gently washed twice with pre‐warmed HBSS (Hanks' Balanced Salt Solution, Gibco) to remove excess dye. For imaging, 1 mL of fresh Imaging Buffer Solution was added to each dish. Fluorescent images were immediately acquired using a Nikon A1 confocal microscope.

### 2.17 Xenograft Mouse Model

Female BALB/c nude mice, aged 4–5 weeks, were purchased from GemPharmatech (Jiangsu, China) and randomly divided into two groups (n = 7 per group). A total of 2 × 10^6^ A549 cells, stably transfected with sh‐scramble or sh‐*GSLR*, were implanted subcutaneously into the nude mice. Tumor growth was monitored weekly, and tumor volume was calculated with the following formula: Volume (cm^3^) = (length × width^2^)/2. After 5 weeks, the mice were euthanized, and the tumors were excised and weighed. All procedures strictly followed the Guide for the Care and Use of Laboratory Animals (NIH publication 80‐23, revised 1996) and were approved by the Animal Ethics Committee of Zhejiang University (No. ZJU20250416).

### 2.18 Statistical Analysis

All quantitative data were presented as mean±standard deviation (SD) from at least three independent biological replicates. Prior to formal statistical comparison, all datasets were first subjected to normality test and homogeneity of variance test to determine the appropriate statistical methods. For comparisons between two experimental groups, statistical significance was determined using two‐tailed unpaired Student's t‐tests with Welch's correction for unequal variances when appropriate. Multiple group comparisons were analyzed using either one‐way ANOVA (for single‐factor experiments) or two‐way ANOVA (for multi‐factorial experimental designs) followed by Tukey's post‐hoc test. The threshold for statistical significance was set at p < 0.05, with the following significance levels indicated throughout the figures: ^*^p < 0.05, ^**^p < 0.01 and ^***^p < 0.001.

## 3 Results

### 3.1 Identification of *GSLR* as a Metabolism‐responsive lncRNA

**FIGURE 1:**
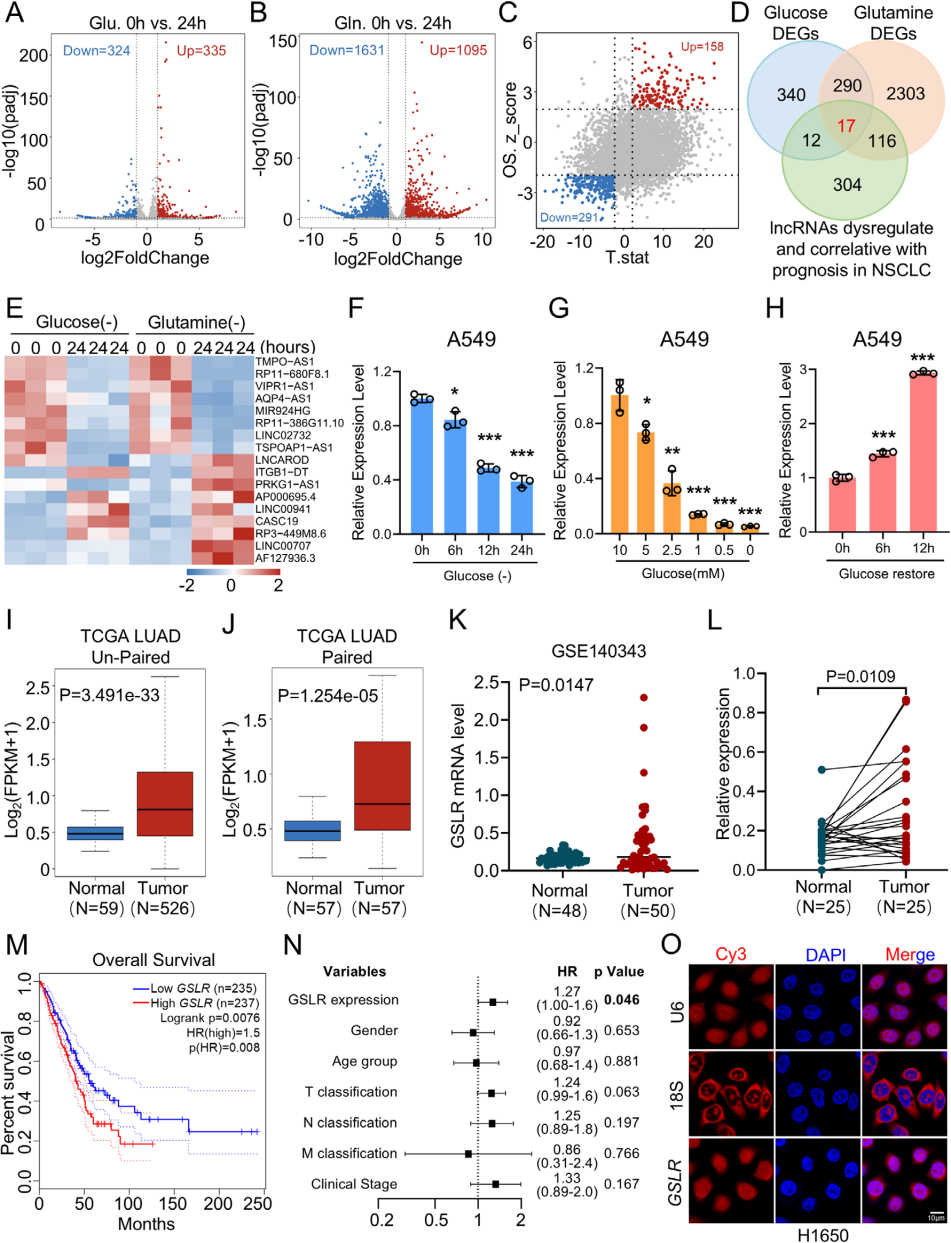
Identification of *GSLR* as a metabolism‐responsive lncRNA. (A, B) Volcano plot showing differentially expressed lncRNAs under glucose (A) or glutamine (B) deprivation (|log2 FoldChange| >1, adjust p‐value <0.05). (C) Differential expression and prognostic relevance of lncRNAs in tumor vs. adjacent tissues from the TCGA‐LUAD dataset. (D) Venn diagram showing the intersection of differentially expressed lncRNAs identified in (A‐C). (E) Heatmap showing expression profiles of 17 candidate lncRNAs under nutrient deprivation. (F‐H) qPCR analysis of *GSLR* expression under glucose deprivation over time (F), across glucose concentrations (G), and during recovery following 24 h deprivation (H). (I‐K) *GSLR* expression in LUAD tissues from TCGA unpaired (I), paired (J), and GEO (GSE140343) (K) datasets. (L) qPCR validation of *GSLR* expression in freshly collected paired lung cancer tissues and adjacent non‐tumor tissues. (M) Kaplan‐Meier analysis of overall survival of lung cancer patients with high vs. low expressions of *GSLR*. (N) Multivariable Cox proportional hazards model assessing the association of *GSLR* expression and clinicopathological characteristics with patient survival. (O) FISH assays showing subcellular localization of *GSLR* in LUAD cells; scale bar, 10 µm. Data are represented as mean±S.D. Statistical significance was determined by one‐way ANOVA followed by Tukey's post‐hoc test (F‐H) or two‐tailed Student's t‐test (I‐L). ^*^ *p* < 0.05, ^**^ *p* < 0.01, ^***^ *p* < 0.001.

Tumor cells typically exhibit anomalously elevated metabolic demands for glucose and glutamine. Therefore, to identify lncRNAs linked to energy metabolism, RNA‐seq was performed on LUAD cells cultured under glucose or glutamine deprivation conditions (Figure 1A, B). By integrating with TCGA‐LUAD dataset (expression and survival data) (Figure 1C), 17 candidate lncRNAs were shortlisted (Figure 1D). Among these, RP11‐680F8.1 (ENST00000536835/NR_135221.1, also termed LCAIIR) demonstrated remarkable sensitivity to both nutrients and was designated as Glu/Gln‐Sensitive LncRNA (*GSLR*) (Figure 1E). *GSLR* is 2588 nucleotides in full length and consists of two exons (Figure S1A). *GSLR* expression decreased progressively with prolonged glucose or glutamine deprivation (Figure 1F, Figure S1B) and with stepwise reductions in glucose or glutamine concentrations (Figure 1G, Figure S1C). Nutrient re‐addition after 24 h deprivation restored *GSLR* expression toward baseline (Figure 1H, Figure S1D). These results indicate that *GSLR* is dynamically regulated by glucose and glutamine in a time‐ and dose‐dependent manner.

We next evaluated the clinical significance of *GSLR*. Analysis of paired and unpaired TCGA‐LUAD samples revealed significantly higher *GSLR* expression in tumor tissues than in adjacent normal tissues (Figure 1I, J). This upregulation was confirmed in an independent GEO dataset (GSE140343) (Figure 1K) and in 25 paired NSCLC specimens from Sir Run Run Shaw Hospital (Figure 1L). Kaplan‐Meier survival analysis showed that high *GSLR* expression was associated with poor overall survival (Figure 1M, N). Fluorescence in situ hybridization (FISH) and nuclear‐cytoplasmic fractionation demonstrated that *GSLR* was predominantly localized in the nucleus (Figure 1O, Figure S1E–G), suggesting a potential transcriptional regulatory role. Additionally, the Coding Potential Calculator 2 (CPC2) classified *GSLR* as a non‐coding RNA (Figure S1H). Taken together, these data identify *GSLR* as a nutrient‐responsive and prognostic lncRNA in LUAD.

### 3.2 Histone Modifications and CTCF Regulate the Transcription of *GSLR*

**FIGURE 2:**
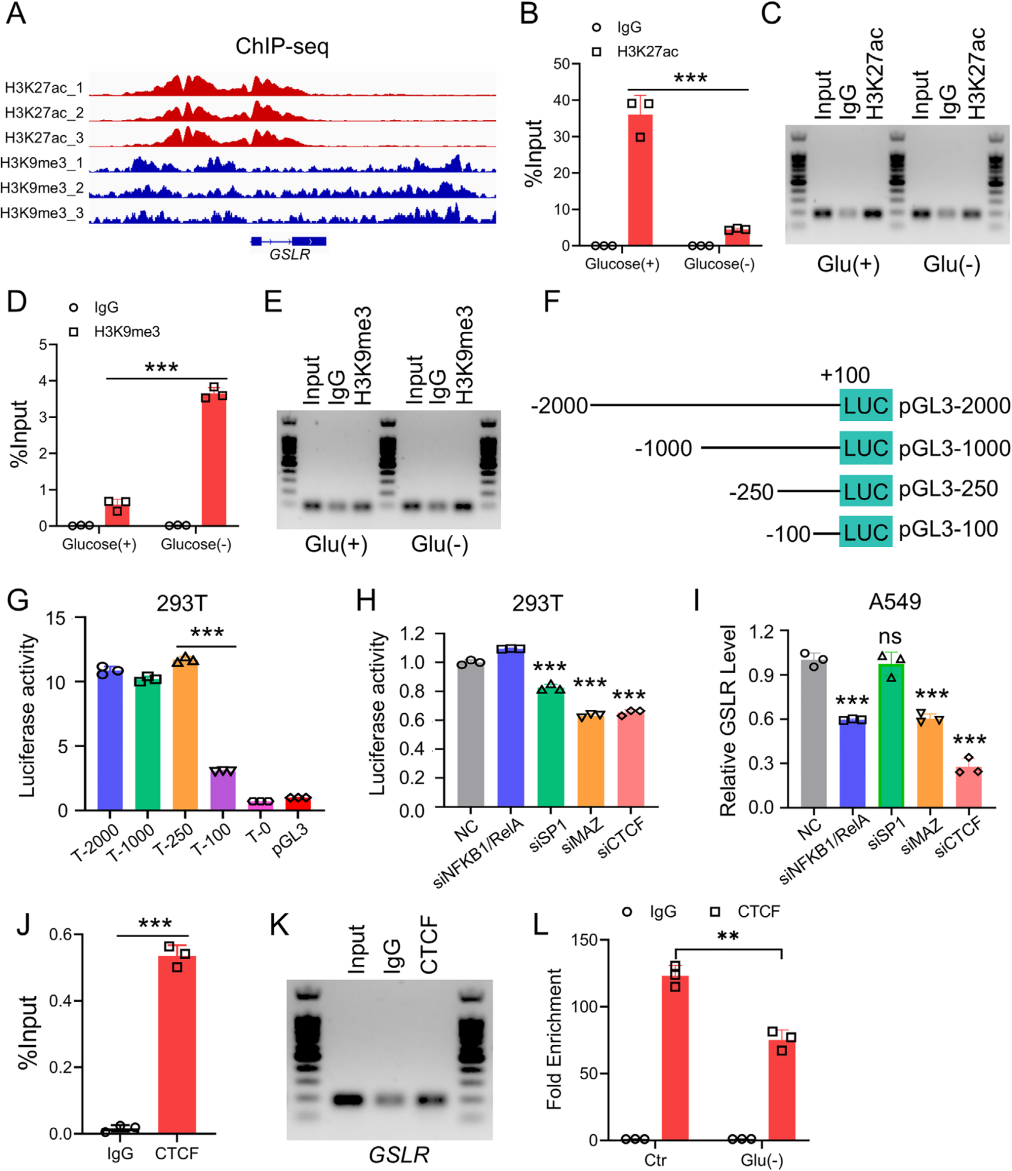
Epigenetic regulation of *GSLR* by histone modifications and CTCF. (A) ChIP‐seq tracks (GSE91337 and GSE91335) showing H3K27ac and H3K9me3 profiles across the *GSLR* genome. (B, C) ChIP‐qPCR and agarose gel electrophoresis showing decreased H3K27ac occupancy at the *GSLR* promoter under glucose deprivation. (D, E) ChIP‐qPCR and agarose gel electrophoresis showing increased H3K9me3 enrichment at the *GSLR* promoter under glucose deprivation. (F) Schematic of the segmented truncation of the *GSLR* promoter (‐2000 bp to +100 bp). (G) Dual‐luciferase reporter assays assessing transcriptional activity of the truncated *GSLR* promoter constructs. (H) Dual‐luciferase assays evaluating the impact of candidate transcription factors on the transcriptional activity of *GSLR*. (I) qPCR assays quantifying the effect of candidate transcription factors on *GSLR* expression in A549 cells. (J, K) ChIP‐qPCR and agarose gel electrophoresis confirming CTCF binding to the *GSLR* promoter. (L) ChIP‑qPCR analysis of CTCF enrichment at the GSLR promoter under normal culture (Ctr) and glucose deprivation (Glu(‐)) conditions. Data are represented as mean±S.D. Statistical significance was determined by two‐tailed unpaired Student's t‐test (B, D, K) or one‐way ANOVA followed by Tukey's post‐hoc test (G‐I), ^***^ *p* < 0.001, ns = not significant.

Given that metabolites serve as substrates or cofactors for chromatin‐modifying reactions [26], we examined epigenetic regulation of *GSLR*. Treatment with the DNA methyltransferase inhibitor 5‐Aza‐2'‐deoxycytidine (5‐Aza‐dC) did not alter *GSLR* expression (Figure S2A, B), suggesting that DNA methylation is not the primary regulatory mechanism. In contrast, nutrient restriction induced substantial remodeling of histone marks, characterized by increased repressive H3K9me3 and H3K27me3 and reduced activating H3K27ac (Figure S2C). ChIP‐seq datasets (GSE91337 and GSE91335) showed distinct peaks of H3K27ac and H3K27me3 at the *GSLR* locus (Figure 2A). Furthermore, ChIP‐qPCR at the *GSLR* promoter under glucose deprivation revealed diminished H3K27ac enrichment (Figure 2B, C) and elevated H3K9me3 occupancy (Figure 2D, E), consistent with a nutrient‐responsive shift in chromatin state at the *GSLR* locus. We next explored the upstream mechanisms underlying this nutrient‑driven histone modification remodeling, by profiling epigenetic enzyme expression and key metabolic cofactor abundance. Western blot analysis revealed that the histone acetyltransferase CBP was markedly downregulated, whereas the histone methyltransferase EZH2 was upregulated under nutrient stress (Figure S2D). In addition, intracellular metabolite quantification showed that glucose deprivation significantly depleted both acetyl‑CoA and α‑ketoglutarate (α‑KG) in A549 cells (Figure S2E, F). Collectively, these findings indicate that nutrient availability dynamically shapes the chromatin landscape to regulate *GSLR* transcription.

To elucidate the *GSLR* transcriptional regulation, we analyzed 2.1 kb promoter region (‐2000 to +100 bp) using luciferase reporter assays. Sequential truncation identified a 150 bp core regulatory region (‐250 to ‐100 bp) with maximal activity (Figure 2F, G). Motif analysis (PROMO/UCSC/JASPAR) predicted five transcription factor binding sites within this region, including NFKB1, RelA, SP1, MAZ, and CTCF. siRNA screening revealed that depletion of MAZ or CTCF significantly reduced promoter activity (Figure 2H), with CTCF knockdown causing the greatest decrease in endogenous *GSLR* expression (Figure 2I, Figure S2G). ChIP‐seq (GSM822289) confirmed CTCF occupancy at the *GSLR* promoter (Figure S2H), which was further validated by ChIP‑qPCR (Figure 2J, K). Moreover, glucose deprivation significantly diminished CTCF enrichment at the *GSLR* promoter (Figure 2L). These findings establish CTCF‐dependent chromatin regulation as a key mechanism controlling *GSLR* transcription.

### 3.3 *GSLR* Promotes Cell Proliferation and Metabolic Remodeling

**FIGURE 3:**
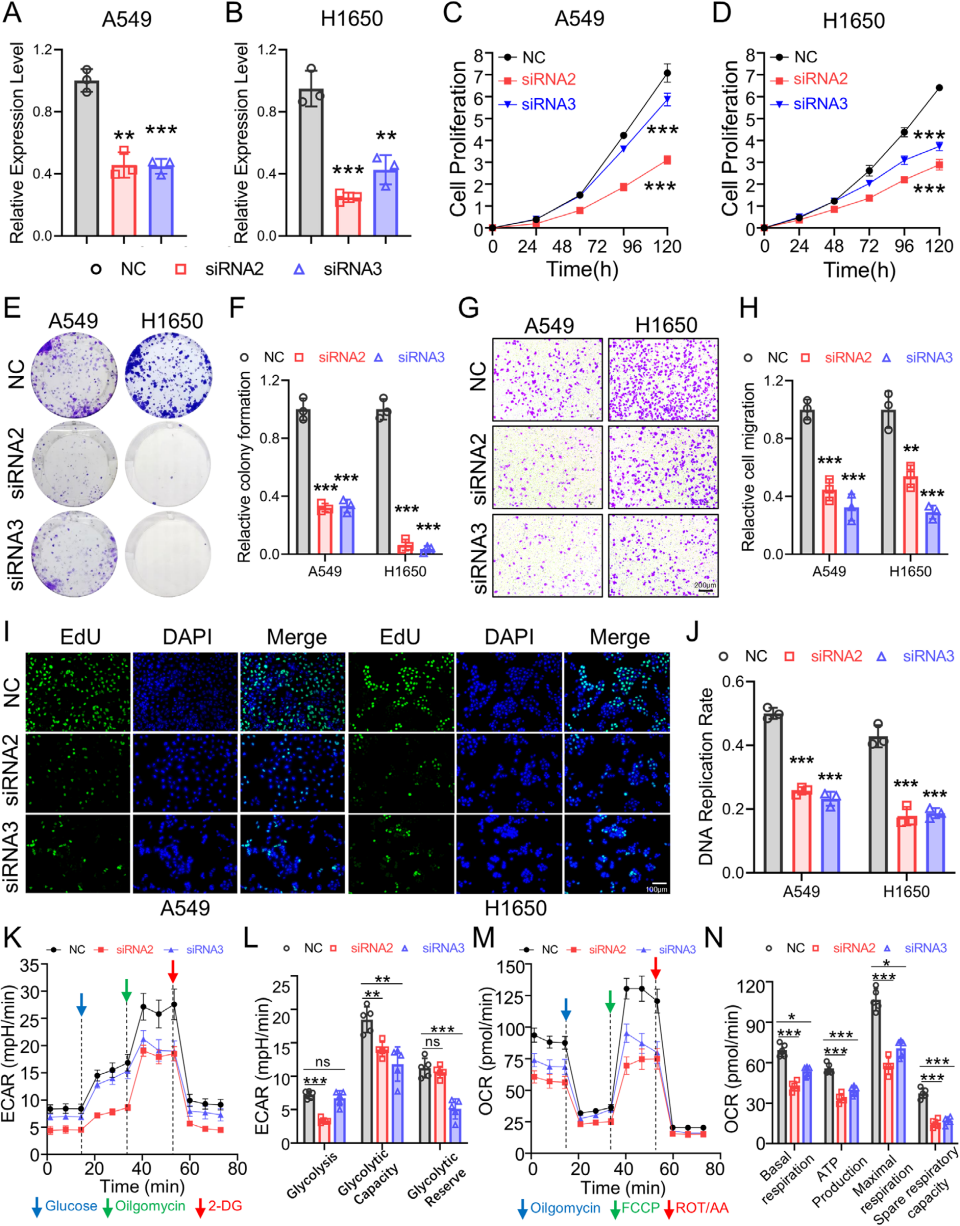
*GSLR* promotes LUAD cell proliferation and metabolic remodeling. (A, B) qPCR analysis of *GSLR* expression in A549 and H1650 cells transfected with two independent siRNAs (si*GSLR*‐2, si*GSLR*‐3) vs. negative control siRNA (siNC). (C, D) CCK‐8 assays assessing cell proliferation in siNC‐ and si*GSLR*‐transfected cells. (E, F) Colony formation assays (E) and quantification of colony number (F) assessing the colony formation abilities in siNC‐ and si*GSLR*‐transfected cells. (G, H) Transwell migration assays (G) and quantification (H) assessing cell migration abilities in siNC‐ and si*GSLR*‐transfected cells; scale bar, 200 µm. (I, J) EdU incorporation assays (I) and quantification (J) assessing DNA‐replication rates in siNC‐ and si*GSLR*‐transfected cells; scale bar, 100 µm. (K, L) ECAR traces from glycolysis stress test and quantification of basal glycolysis, maximum glycolytic capacity, and glycolytic reserve. (M, N) OCR traces from mitochondrial stress test and quantification of basal respiration, ATP production, maximal respiration, and spare respiratory capacity. Data are represented as mean±S.D. Statistical significance was determined by one‐way ANOVA followed by Tukey's post‐hoc test (A‐D, F, H, J, L, N), ^*^ *p* < 0.05, ^**^p < 0.01, ^***^ *p* < 0.001, ns = not significant.

To explore the biological function of *GSLR* in LUAD, we designed two specific siRNAs to efficiently silence *GSLR* expression in A549 and H1650 cells (Figure 3A, B). *GSLR* knockdown significantly reduced cell proliferation in CCK8 assays (Figure 3C, D) and colony‐forming ability in colony formation assays (Figure 3E, F), diminished migration ability in Transwell assays (Figure 3G, H), and decreased DNA replication in EdU assays (Figure 3I, J). We further generated LUAD cells with stable knockdown or overexpression (Figure S3A, E). Consistent with transient knockdown, stable *GSLR* depletion suppressed cell proliferation (Figure S3B, C) and colony formation (Figure S3D), whereas *GSLR* overexpression enhanced these malignant phenotypes (Figure S3F–H).

Given the central role of glycolysis and oxidative phosphorylation in cellular energy metabolism, we next investigated whether *GSLR* contributes to metabolic regulation. Extracellular acidification rate (ECAR) and oxygen consumption rate (OCR) assays demonstrated that *GSLR* knockdown significantly attenuated glycolytic capacity (Figure 3K, L) and impaired oxidative phosphorylation efficiency (Figure 3M, N). Taken together, these findings indicate that *GSLR* promotes LUAD cell growth and migration while supporting metabolic reprogramming.

### 3.4 *GSLR* Directly Interacts With DHX9 in LUAD Cells

**FIGURE 4:**
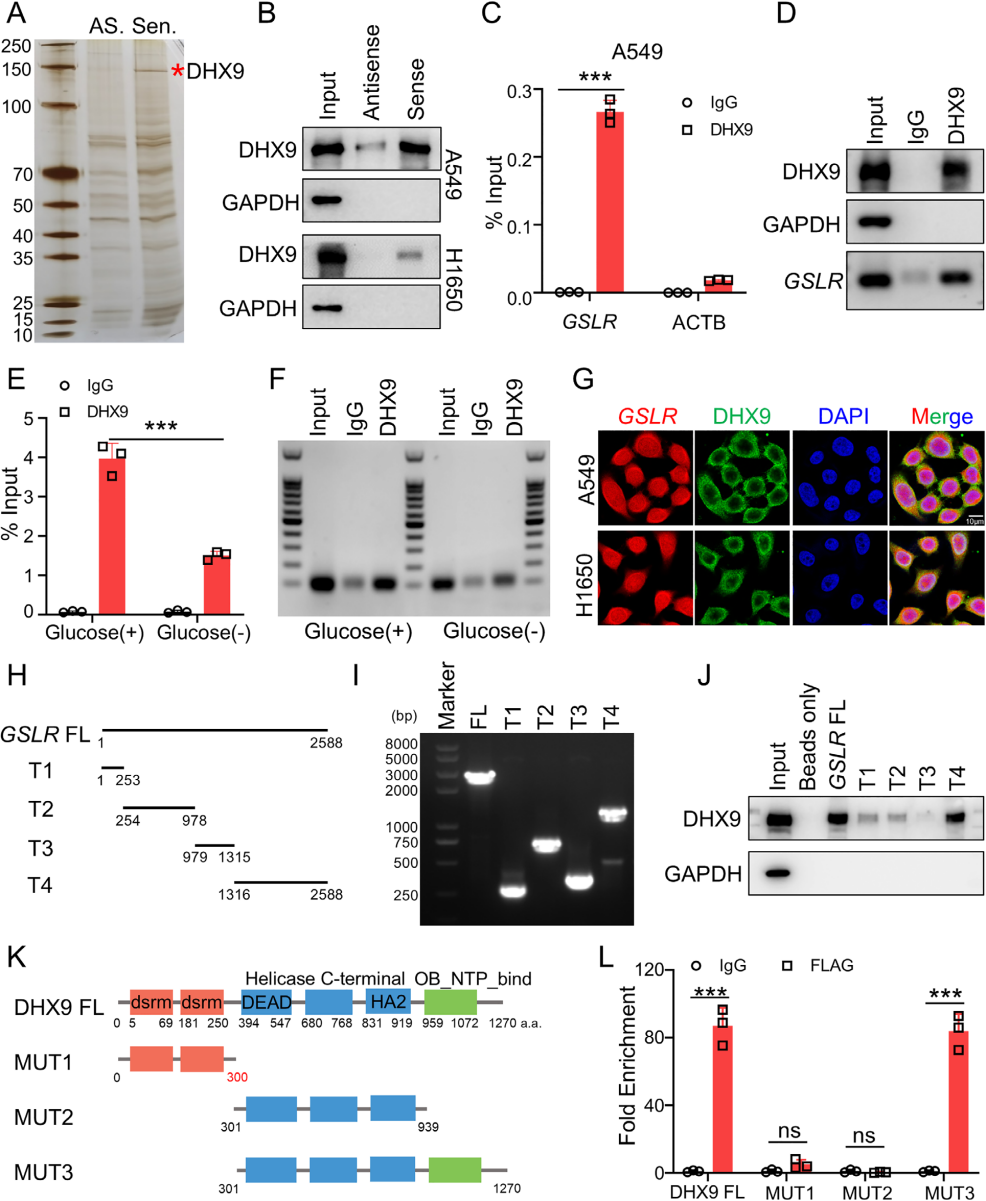
*GSLR* directly associates with DHX9. (A) Proteins interacting with *GSLR* identified by pull down assay followed by mass spectrometry. (B) RNA pull‐down and western blot assays confirmed the *GSLR*‐DHX9interaction in A549 and H1650 cell lines. (C) RIP assays showing enrichment of *GSLR* in DHX9 immunoprecipitates; ACTB served as a negative control. (D) Western blot and agarose gel electrophoresis assessing DHX9 immunoprecipitation efficiency and *GSLR* enrichment, respectively. (E, F) RIP assays and agarose gel electrophoresis showing reduced *GSLR* enrichment by DHX9 under glucose deprivation. (G) Immunofluorescence image showing subcellular localization of *GSLR* and DHX9 in A549 and H1650 cells; scale bar, 10 µm. (H) Schematic of full‐length and truncated mutants (T1‐4) used for mapping *GSLR*‐binding regions. (I) Agarose gel electrophoresis images of *GSLR* full‐length and truncated mutants. (J) RNA pull‐down assays assessing the binding efficiency of full‐length *GSLR* and truncated mutants to DHX9; beads alone served as a negative control. (K) Schematic of full‐length DHX9 and truncated mutants (MUT1‐4). (L) RIP assays measuring enrichment of *GSLR* by FLAG‐tagged full‐length DHX9 protein and domain mutants. Data are represented as mean±S.D. Statistical significance was determined by two‐tailed unpaired Student's t‐test (C, E, L), ^***^ *p* < 0.001, ns = not significant.

To investigate the molecular mechanisms of *GSLR* in LUAD, we performed RNA pull‐down assays using in vitro transcribed sense and antisense *GSLR* RNAs. Silver staining of the pull‐down products revealed a prominent band at approximately 150 kDa, which was specifically associated with sense *GSLR* (Figure 4A). This specific band was excised and subjected to mass spectrometry, which identified DHX9 as a binding protein for *GSLR* (Figure S4A). Western blotting confirmed stable *GSLR*‐DHX9 interaction in A549 and H1650 cells (Figure 4B). RIP assays further validated this association, showing significant enrichment of *GSLR* in DHX9 immunoprecipitates compared with controls (Figure 4C, D). Notably, glucose deprivation markedly weakened DHX9 binding to *GSLR* (Figure 4E, F, Figure S4B), suggesting the metabolic regulation of this complex. Immunofluorescence further revealed strong colocalization of *GSLR* and DHX9 (Figure 4G), supporting their functional linkage.

To map the regions that mediate the interaction between *GSLR* and DHX9, we subdivided *GSLR* into four structural segments as predicted by RNAfold (Figure 4H, I, Figure S4C). Pull‐down assays using in vitro synthesized full‐length (FL) *GSLR* and T1 (1‐253), T2 (254‐978), T3 (979‐1315), and T4 (1316‐2588) fragments were performed, and the products were analyzed using western blotting. We demonstrated that the T4 fragment could bind to DHX9, whereas the other fragments and the beads‐only control could not (Figure 4J). On the other hand, DHX9 contains two N‐terminal dsRBDs, a helicase core domain, and a C‐terminal OB‐fold [27, 28]. Then, we constructed four human FLAG‐tagged DHX9 vectors harboring FL (1‐1270), MUT1 (1‐300), MUT2 (301‐939) and MUT3 (301‐1270) to identify the DHX9 residues that associated with *GSLR* (Figure 4K). RIP assays using an anti‐FLAG antibody or IgG control showed that the OB‐fold domain was the primary *GSLR*‐binding region (Figure 4L, Figure S4D, E). Together, these findings indicate a specific interaction between the central region (1316‐2588 nt) of *GSLR* and the C‐terminal OB‐fold domain of DHX9, forming a metabolically regulated nuclear complex.

### 3.5 DHX9 Promotes LUAD Cell Proliferation and Metastasis

**FIGURE 5:**
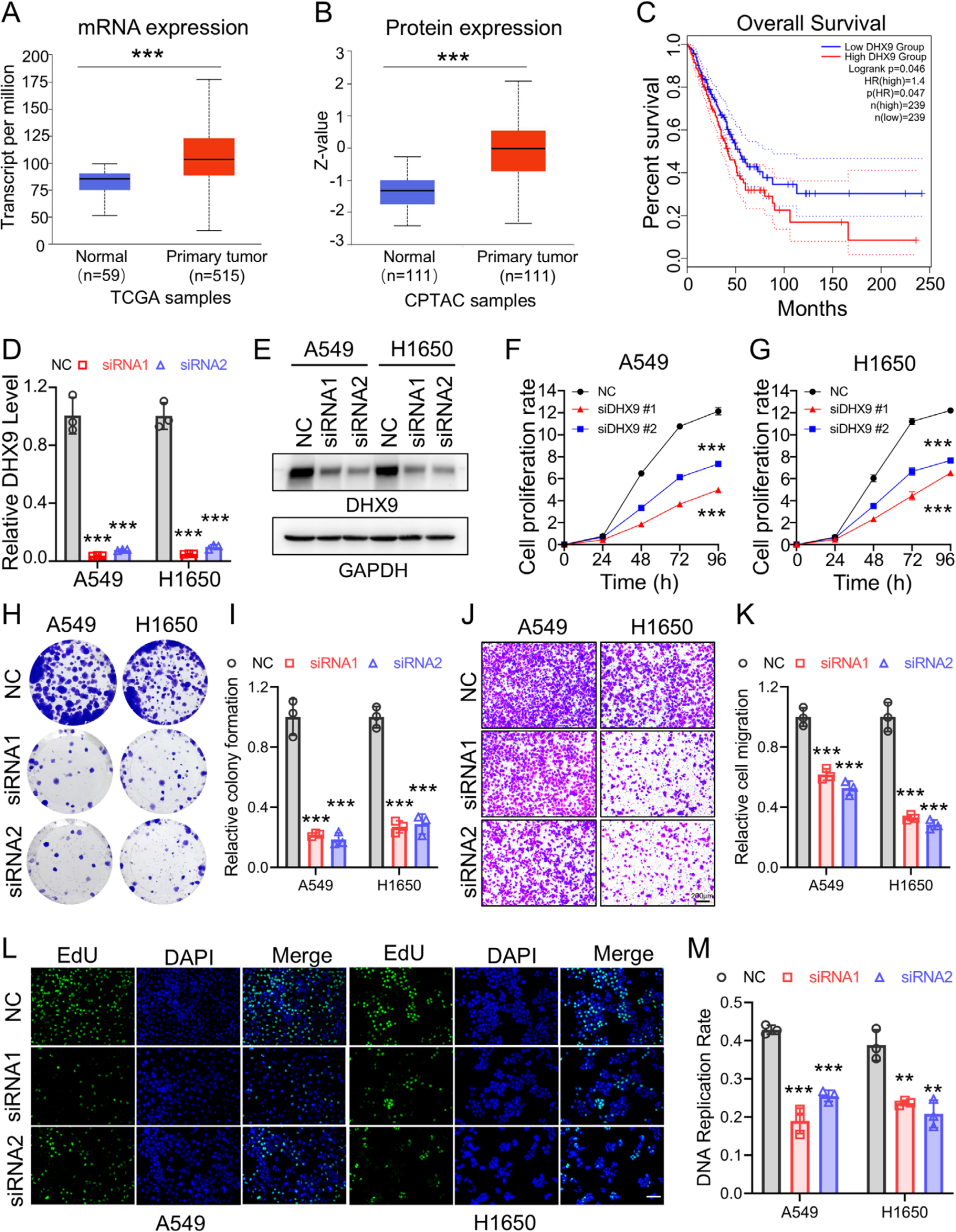
DHX9 promotes proliferation and metastasis in LUAD cells. (A) DHX9 mRNA expression in primary tumors (n = 515) and adjacent normal tissues (n = 59) from TCGA LUAD datasets. (B) DHX9 protein expression in primary tumors (n = 111) and adjacent normal tissue (n = 111) from CPTAC LUAD dataset. (C) Kaplan‐Meier analysis of overall survival of LUAD patients with high vs. low DHX9 expressions. (D) qPCR analysis of DHX9 mRNA levels in A549 and H1650 cells transfected with DHX9 siRNA (siDHX9#1 or siDHX9#2) and negative control siRNA (siNC). (E) Western blot analysis of DHX9 protein levels in A549 and H1650 cells transfected with siNC or siDHX9. (F, G) CCK‐8 assays assessing cell proliferation in siNC‐ and siDHX9‐transfected lung cancer cells. (H, I) Representative colony formation images (H) and quantification of colony numbers (I) in siNC‐ and siDHX9‐transfected lung cancer cells. (J, K) Transwell migration assays (J) and quantification (K) comparing migration of siNC‐ and siDHX9‐transfected lung cancer cells; scale bar, 200 µm. (L, M) EdU incorporation assays (L) and quantification (M) showing reduced DNA replication after DHX9 knockdown; scale bar, 100 µm. Data are represented as mean±S.D. Statistical significance was determined by one‐way ANOVA followed by Tukey's post‐hoc test (D, F, G, I, K, M). ^**^ *p* < 0.01, ^***^ *p* < 0.001.

DHX9, a multifunctional RNA helicase involved in transcriptional regulation, genome stability maintenance, and RNA processing [29, 30], was significantly upregulated in LUAD tissues compared to normal controls, as shown by TCGA and CPTAC analyses (Figure 5A, B). This finding was validated in seven paired NSCLC specimens from Sir Run Run Shaw Hospital, where DHX9 protein levels were consistently elevated in tumors (Figure S5A). Kaplan‐Meier survival analysis revealed that high DHX9 expression correlated with poorer overall survival (Figure 5C), supporting an oncogenic role in LUAD.

To further assess the biological function of DHX9, we performed siRNA‐mediated DHX9 knockdown in A549 and H1650 cells, which was verified using qPCR and western blotting (Figure 5D, E). CCK8 assays showed that DHX9 depletion significantly inhibited cell proliferation (Figure 5F, G). Colony formation assays confirmed that DHX9 knockdown significantly reduced colony formation ability (Figure 5H, I). Transwell assays showed that DHX9 knockdown significantly reduced cell migration (Figure 5J, K). EdU incorporation assays showed that DHX9 knockdown inhibited DNA replication activity (Figure 5L, M). Similar defects were observed in stable DHX9‐depleted cells generated using shRNA (Figure S5B‐E). Together, these loss‐of‐function experiments established DHX9 as a key driver of LUAD cell growth and metastatic potential.

Next, we generated full‐length DHX9 (DHX9 FL) and its binding‐deficient mutant (DHX9 ΔOB‐fold). CCK‐8 and colony formation assays showed that overexpression of DHX9 FL significantly enhanced cell proliferation and clonogenicity, whereas overexpression of DHX9 ΔOB‐fold largely abrogated these effects (Figure S5F–H). Consistent results were obtained with *GSLR* FL and its binding‐deficient mutant *GSLR* ΔT4 (Figure S5I–K). Collectively, these findings establish that the physical interaction between *GSLR* and DHX9 is essential for their oncogenic function in LUAD.

### 3.6 CKB Is a Downstream Target of the *GSLR*‐DHX9 Complex

**FIGURE 6:**
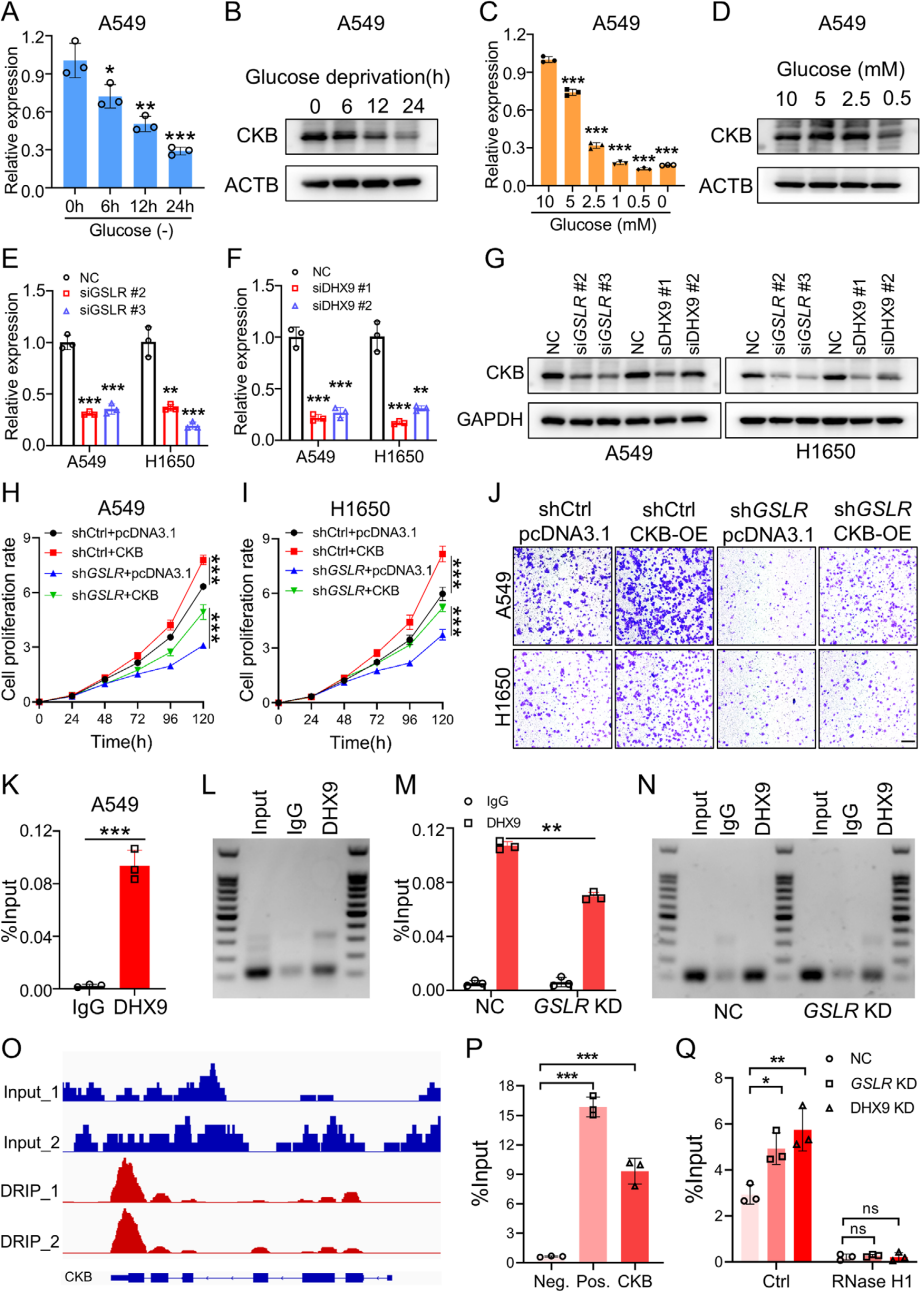
CKB is a downstream target of the *GSLR*/DHX9 complex. (A, B) qPCR and western blot analysis of CKB expression over time (0‐24 h) under glucose deprivation. (C, D) qPCR and western blot analysis of CKB expression in cells exposed to varying glucose concentrations (0‐10 mM) for 24 h. (E, F) qPCR analysis of CKB mRNA levels following *GSLR* or DHX9 knockdown. (G) Western blot analysis of CKB protein levels after *GSLR* or DHX9 knockdown in A549 and H1650 cells. (H, I) CCK‐8 assays assessing cell proliferation in A549 and H1650 cells transfected with shCtrl+pcDNA3.1, shCtrl+CKB‐OE, sh*GSLR*+pcDNA3.1, or sh*GSLR*+CKB‐OE. (J) Transwell assays assessing cell migration ability in A549 and H1650 cells transfected with shCtrl+pcDNA3.1, shCtrl+CKB‐OE, sh*GSLR*+pcDNA3.1, or sh*GSLR*+CKB‐OE. Scale bar: 200 µm. (K, L) ChIP‐qPCR and agarose gel electrophoresis showing the binding efficiency of DHX9 to the CKB promoter. (M, N) ChIP‐qPCR and agarose gel electrophoresis showing reduced DHX9 occupancy at the CKB promoter following *GSLR* knockdown. (O) DRIP‐seq track (GSE300469) showing R‐loop enrichment across the CKB gene. Two independent biological replicates are shown; blue peaks representing input control signals; red peaks representing R‐loop signals. (P) DRIP‐qPCR confirming R‐loop formation at the CKB locus. EGR1served as a negative control and RPL13A as a positive control. (Q) DRIP‐qPCR analysis of R‐loop levels at the CKB locus in A549 cells with or without RNase H1 treatment, under NC, *GSLR* KD, or DHX9 KD conditions. R‐loop levels were quantified using the %Input method. Data are represented as mean±S.D. Statistical significance was determined by one‐way ANOVA followed by Tukey's post‐hoc test (A, C, E, F, H, I, P, Q) or two‐tailed unpaired Student's t‐test (K, M). ^*^ *p* < 0.05, ^**^ *p* < 0.01, ^***^ *p* < 0.001, ns = not significant.

To identify genes co‐regulated by *GSLR* and DHX9, we analyzed RNA‐seq datasets from glucose deprivation, glutamine deprivation, and knockdown *GSLR* or DHX9 (Figure S6A–D). Cross‐comparison revealed 36 shared differentially expressed genes, including nine consistently downregulated and five upregulated genes (Figure S6E, F). Among these, creatine kinase type B (CKB), a key regulator of cellular energy homeostasis [31], was significantly suppressed under all four conditions. qPCR and western blot analyses confirmed that CKB mRNA and protein levels were reduced by glucose or glutamine deprivation in a time‐ and dose‐dependent manner (Figure 6A–D, Figure S6G–J). Similarly, silencing *GSLR* or DHX9 decreased CKB at both mRNA and protein levels (Figure 6E–G). Notably, dual knockdown did not further reduce CKB expression (Figure S6K–M), suggesting that *GSLR* and DHX9 synergistically regulate CKB expression. Furthermore, qPCR analyses showed that overexpression of full‐length *GSLR* or DHX9 significantly upregulated CKB mRNA levels. In contrast, the binding‐deficient mutants (*GSLR* ΔT4 and DHX9 ΔOB‐fold) completely lost the ability to induce CKB expression (Figure S6N, O). These results determined that this regulation requires the physical interaction between *GSLR* and DHX9.

Although CKB has been implicated in energy metabolism, ferroptosis and tumor progression [32, 33], its role in LUAD remains unclear. Therefore, we generated LUAD cell lines with CKB knockdown or overexpression (Figure S7A, G). CKB depletion significantly inhibited cell proliferation (Figure S7B, C), colony formation (Figure S7D), and DNA synthesis (Figure S7E, F), whereas CKB overexpression enhanced these malignant phenotypes (Figure S7H–J), indicating that CKB promotes LUAD progression.

To determine whether *GSLR* exerts its oncogenic functions through upregulating CKB, we performed functional rescue experiments by overexpressing CKB in *GSLR*‐stable‐knockdown A549 and H1650 cells. CCK‐8 assays showed that *GSLR* knockdown significantly inhibited cell proliferation, while exogenous overexpression of CKB largely reversed this inhibitory effect (Figure 6H, I). Consistently, Transwell migration assays demonstrated that the reduced migration ability caused by *GSLR* depletion was also largely rescued by CKB overexpression (Figure 6J, Figure S7K). These results confirmed that CKB is the key functional downstream effector mediating *GSLR*‐induced LUAD cell proliferation and migration.

Given that *GSLR* and DHX9 are mainly located in nucleus, we investigated whether the *GSLR*‐DHX9 complex directly regulates CKB transcription. ChIP‐qPCR revealed DHX9 binding at the CKB promoter (Figure 6K, L), which was diminished by *GSLR* knockdown (Figure 6M, N). Consistently, glucose deprivation similarly reduced DHX9 occupancy at the CKB promoter (Figure S7L, M), indicating that nutrient stress modulates CKB transcription via the *GSLR*‐DHX9 axis. DHX9 has been reported to resolve R‐loops [28, 34, 35]. DRIP‐seq revealed R‐loop formation within the CKB locus (Figure 6O). Consistent with this finding, DRIP‐qPCR confirmed R‐loop enrichment at CKB (Figure 6P). Furthermore, *GSLR* or DHX9 knockdown significantly increased R‐loop enrichment at the CKB gene locus. However, RNase H1 treatment abolished R‐loop accumulation in all three groups (Figure 6Q). These findings suggest that *GSLR* recruits DHX9 to the CKB promoter to prevent R‐loop accumulation and facilitate transcription.

### 3.7 The *GSLR*‐DHX9‐CKB Axis Regulates Mitochondrial‐dependent Apoptosis

**FIGURE 7:**
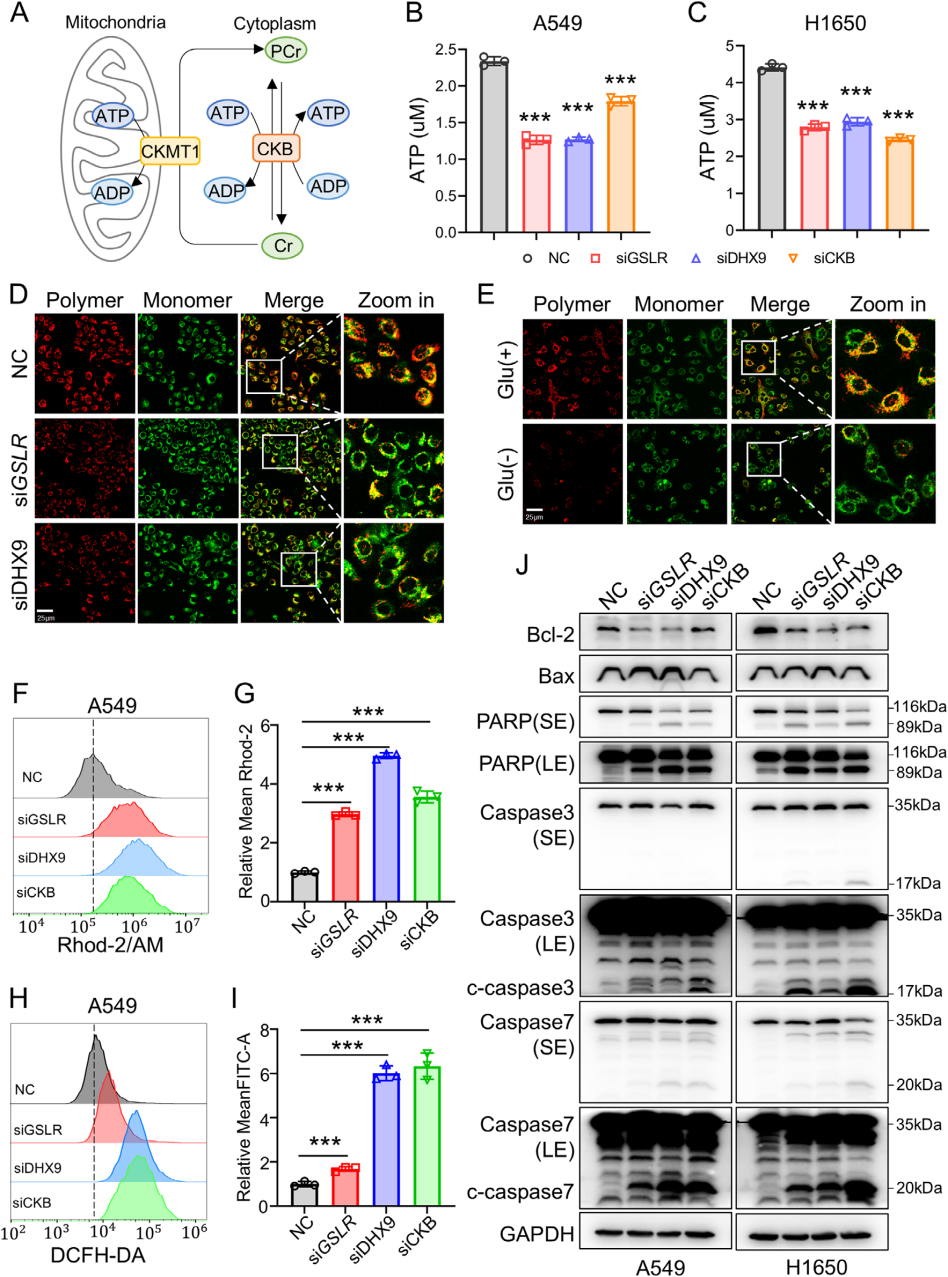
The *GSLR*‐DHX9‐CKB axis regulates mitochondrial‐dependent apoptosis. (A) Schematic of the cellular energy shuttle system of creatine kinase/phosphocreatine. PCr: phosphocreatine; Cr: creatine kinase. (B, C) ATP production in A549 and H1650 cells following knockdown of *GSLR*, DHX9, or CKB. (D) Confocal images of JC‐1 staining showing mitochondrial membrane potential (ΔΨm) in H1650 cells after *GSLR* or DHX9 knockdown; scale bar, 25 µm. (E) JC‐1 staining showing ΔΨm changes in A549 cells under glucose deprivation; scale bar, 25 µm. (F) Flow cytometry analysis of mitochondrial Ca^2+^ levels in A549 cells following knockdown of *GSLR*, DHX9, or CKB using 4 µM Rhod‐2/AM probe for 30 min. (G) Quantification of Rhod‐2 fluorescence intensity in (F) from three independent experiments. (H) Flow cytometry analysis of intracellular ROS levels in A549 cells after knockdown of *GSLR*, DHX9, or CKB using 10 µM DCFH‐DA probe for 20 min. (I) Quantification of DCF fluorescence intensity in (H) from three independent experiments. (J) Western blot analysis of apoptosis‐related proteins following knockdown of *GSLR*, DHX9, or CKB; SE: short exposure; LE: long exposure. Data are represented as mean±S.D. Statistical significance was determined by one‐way ANOVA followed by Tukey's post‐hoc test (B, C, G, I), ^***^ *p* < 0.001.

CKB maintains cellular energy homeostasis by catalyzing phosphate transfer between phosphocreatine and ATP [32, 36] (Figure 7A). Disruption of the *GSLR*/DHX9/CKB axis significantly reduced intracellular ATP levels (Figure 7B, C), underscoring its role in energy maintenance. Mitochondria are the primary site of cellular ATP production. Previous studies have shown that CKB enhances mitochondrial ATP synthesis by inhibiting activation of the mitochondrial permeability transition pore (mPTP) [37]. We therefore assessed mitochondrial function using the JC‐1 fluorescent probe. Knockdown of *GSLR* or DHX9 sharply reduced the mitochondrial membrane potential (ΔΨm) (Figure 7D, Figure S8A) and similar ΔΨm loss was observed under glucose deprivation (Figure 7E, Figure S8B), indicating impaired mitochondrial efficiency.

We used flow cytometry with Rhod‐2, a high‐affinity mitochondrial Ca^2+^ indicator, to measure intracellular calcium levels. Knockdown of *GSLR*, DHX9, or CKB all significantly increased mitochondrial calcium concentrations (Figure 7F, G). These results indicate that loss of ΔΨm triggers mPTP opening, which in turn causes mitochondrial Ca^2^^+^ overload. Furthermore, DCFH‐DA‐based ROS measurements revealed increased intracellular ROS accumulation under glucose deprivation (Figure S8C, D) and after silencing of *GSLR*, DHX9, or CKB (Figure 7H, I, Figure S8E, F), indicating oxidative stress triggered by metabolic disruption.

Persistent mitochondrial dysfunction can induce intrinsic apoptosis [38]. Flow cytometric analysis with Annexin V‐FITC/PI staining revealed increased early apoptosis in cells with *GSLR*, DHX9, or CKB knockdown (Figure S8G). Western blot analysis further showed a marked downregulation of the anti‐apoptotic protein Bcl‐2 as well as a significant upregulation of cleaved‐caspase3, cleaved‐caspase7 and cleaved‐PARP (Figure 7J). Collectively, these findings demonstrated that the *GSLR*‐DHX9 axis controls CKB transcription to sustain mitochondrial integrity and energy metabolism; disruption of this axis causes ΔΨm loss, Ca^2^^+^ overload, ROS accumulation, and activation of the caspase cascade.

### 3.8 Effects of *GSLR* on LUAD Tumor Growth and Metastasis In Vivo

**FIGURE 8:**
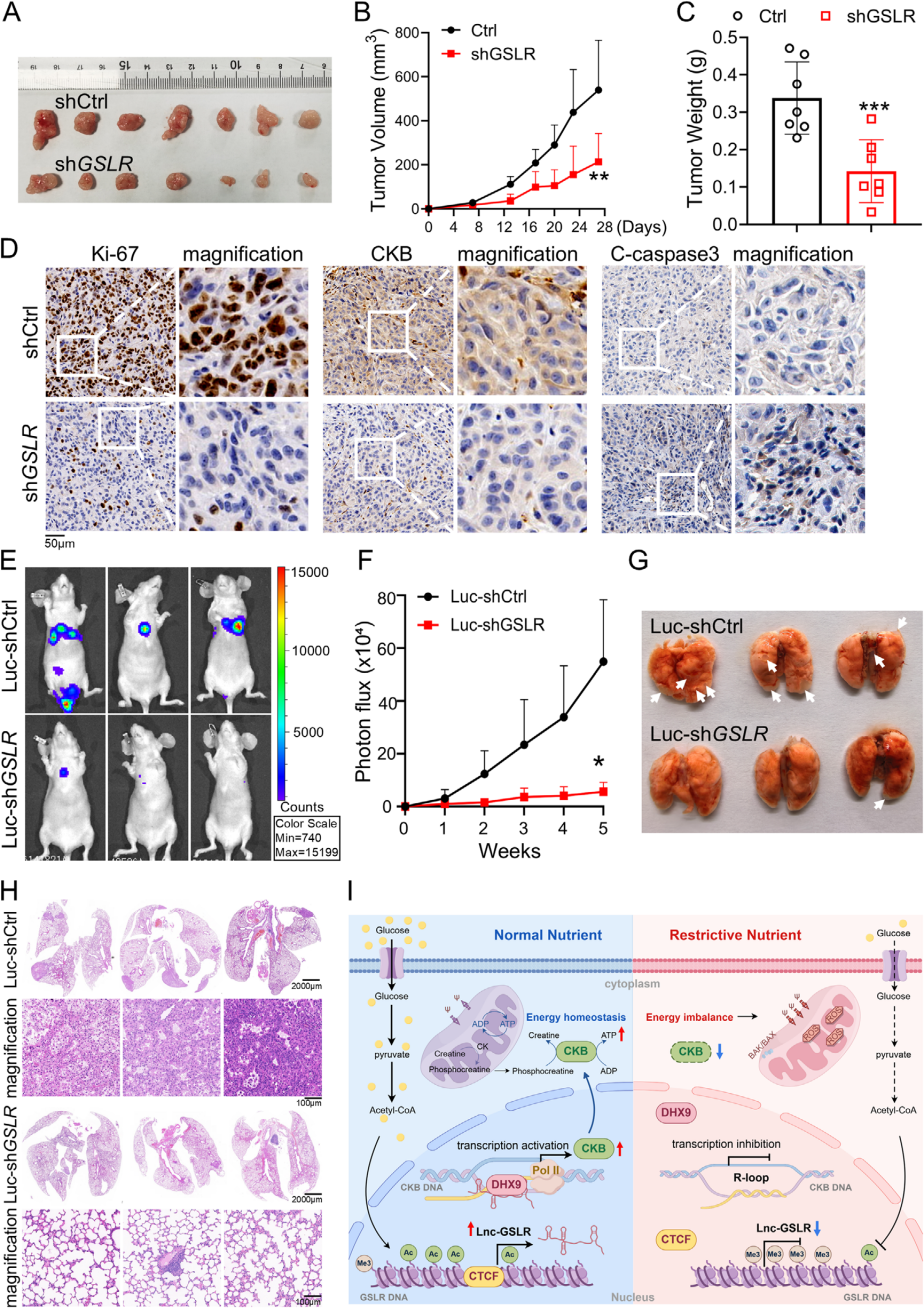
*GSLR* promotes LUAD tumor growth and metastasis in vivo. (A) Image of xenograft tumors resected from nude mice injected subcutaneously with A549 cells expression sh‐*GSLR* or sh‐Ctrl (n = 7). (B) Tumor volume measured weekly after cell injection (n = 7). (C) Tumor weight measured at the endpoint after tumor resection (n = 7). (D) Representative IHC staining of Ki‐67, CKB and cleaved‐caspase3 in xenograft tumor sections sh‐Ctrl and sh‐*GSLR* groups; scale bar, 50 µm. (E, F) Representative bioluminescence images and statistical analysis of lung metastases in mice via tail vein injection of A549‐Luc‐shCtrl and A549‐Luc‐sh*GSLR* cells (n = 3). (G, H) Representative lung images (G) at week 5, corresponding hematoxylin eosin‐stained lung sections are shown (H). Arrows, lung metastasis foci. (I) Working model showing how the lncRNA *GSLR* regulates cellular energy homeostasis via the DHX9‐CKB axis. Data are represented as mean±S.D. Statistical significance was determined by two‐tailed unpaired Student's t‐test (B, C, F). ^*^p < 0.05, ^**^ *p* < 0.01, ^***^ *p* < 0.001.

To explore the role of *GSLR* in tumorigenesis in vivo, we established stable A549 cell lines with *GSLR* knockdown and implanted them subcutaneously into nude mice. *GSLR* knockdown significantly reduced tumor volume and weight compared with control xenografts (Figure 8A–C). Immunohistochemistry showed *GSLR* knockdown markedly decreased the expression of the proliferation marker Ki‐67 and the downstream target CKB, while significantly increasing the expression of the apoptosis marker cleaved caspase‐3 (Figure 8D), consistent with the in vitro findings. Moreover, we investigated the role of *GSLR* in LUAD metastasis in vivo. A549‐Luc‐shCtrl and A549‐Luc‐sh*GSLR* cells were injected into the tail veins of nude mice respectively. As shown by bioluminescence imaging, A549‐Luc‐shCtrl cells effectively metastasized to the lung region of nude mice at 5 weeks, whereas A549‐Luc‐sh*GSLR* cells did not (Figure 8E, F). Pulmonary anatomy and H&E staining showed that *GSLR* knockdown resulted in fewer metastatic foci in lungs than the control group (Figure 8G, H). Collectively, these findings demonstrated that *GSLR* promotes LUAD growth and metastasis in vivo.

## 4 Discussion

Metabolic reprogramming is a hallmark of malignant tumors [5, 39], with glucose and glutamine serving as essential carbon and nitrogen sources that support proliferation and survival [40, 41]. Under nutrient restriction, tumor cells rewire gene expression networks to sustain metabolic homeostasis [42, 43], and lncRNAs serve as critical regulators of this adaptive response via diverse mechanisms [14, 44, 45, 46]. Here, by integrating RNA‐seq data from glucose/glutamine‐deprived LUAD cells with TCGA datasets, we identified *GSLR* as an energy stress‐responsive lncRNA in LUAD. *GSLR* expression was dynamically regulated by glucose and glutamine in a time‐ and dose‐dependent manner. Clinically, *GSLR* was significantly upregulated in LUAD tissues and correlated with poor prognosis, supporting its potential as a diagnostic and prognostic biomarker and a candidate therapeutic target.

Metabolic fluctuations shape gene expression partly by modulating the activity and substrate availability of epigenetic enzymes [26]. In this study, nutrient restriction triggered chromatin remodeling at the *GSLR* locus, which was marked by increased repressive histone modifications (H3K9me3 and H3K27me3) and reduced activating mark H3K27ac, consistent with a shift toward transcriptional repression. Promoter analysis, luciferase assays, and ChIP‐qPCR further identified CTCF as a key transcriptional activator of *GSLR*, directly binding its promoter to enhance its expression. Functionally, *GSLR* promoted LUAD cell growth and metastasis both in vitro and in vivo. Seahorse analysis revealed that *GSLR* knockdown impaired both glycolysis and oxidative phosphorylation, indicating that *GSLR* facilitates metabolic reprogramming to sustain LUAD cell growth.

Mechanistically, we identified DHX9, an RNA helicase involved in R‐loop resolution and antiviral immunity [35, 47], as a direct binding partner of *GSLR*. The interaction between *GSLR* and DHX9 was attenuated under glucose deprivation, suggesting metabolic regulation of this lncRNA‐protein complex. Mapping experiments revealed that nucleotides 1316–2588 of *GSLR* and the C‐terminal OB‐fold domain of DHX9 mediate this interaction. Notably, as a multifunctional RNA‑binding protein, DHX9 interacts with a broad repertoire of endogenous transcripts [48, 49, 50, 51, 52]. We demonstrated that GSLR is expressed at sufficient abundance in LUAD cells to support a physiological, specific interaction with DHX9, rather than non‑specific spurious binding (Figure S9A–C). Furthermore, the GSLR–DHX9 association does not globally disrupt DHX9 interactions with other endogenous RNAs (Figure S9D), supporting the target gene specificity of this regulatory axis.

Given the predominantly nuclear localization of both *GSLR* and DHX9, we explored downstream targets and identified CKB, a key enzyme in ATP regeneration, as the major effector of the *GSLR–*DHX9 complex. Beyond its canonical role in energy homeostasis, CKB is increasingly recognized as a regulator of tumor biology [33, 53]. In hepatocellular carcinoma cells, CKB phosphorylates and stabilizes GPX4, thereby abrogating ferroptosis and promoting tumor growth [32]. In glioblastoma stem cells, CKB‐derived phosphocreatine modulates epigenetic regulation by preventing BRD2 degradation and ensuring accurate chromosome segregation [54]. However, the role and regulation of CKB in lung cancer remain poorly understood. Although several studies have indicated that CKB is a poor prognostic factor for lung cancer patients and correlates positively with tumor metastasis [53, 55], the underlying molecular mechanisms driving its oncogenic function in lung cancer remain largely uncharacterized. Here we showed that CKB promoted LUAD cell proliferation and migration. Nutrient deprivation and knockdown of either *GSLR* or DHX9 reduced CKB expression, indicating a coordinated regulation. ChIP assays confirmed DHX9 binding at the CKB promoter, and this binding was diminished upon *GSLR* silencing. Disruption of DHX9 recruitment led to increased R‐loop accumulation at the CKB locus and transcriptional repression, consistent with the role of DHX9 in R‐loop resolution. Functionally, the inactivation of the *GSLR*‐DHX9‐CKB axis impaired mitochondrial function, as evidenced by reduced membrane potential, Ca^2^^+^ overload, and elevated ROS levels. These changes ultimately triggered the intrinsic apoptosis, as shown by increased AnnexinV staining and activation of caspase‐7 and PARP cleavage. Thus, this axis supports tumor cell survival by maintaining mitochondrial integrity and energy homeostasis.

While our study focused on glucose and glutamine deprivation, we speculate that the *GSLR*‐DHX9‐CKB axis may also respond to other metabolic challenges in the tumor microenvironment. Hypoxia, lipid deprivation, and oxidative stress are well‑documented to trigger extensive metabolic reprogramming in cancer cells, and lncRNAs have emerged as critical mediators of these adaptive responses [22, 56]. For instance, hypoxia inducible factor 1α (HIF‐1α), a master regulator of hypoxic adaptation, has been reported to activate multiple lncRNAs, including *SZT2‐AS1*, *PMAN*, and *HILPS*, to facilitate tumor metabolic rewiring [22, 57, 58]. Future studies should investigate whether *GSLR* is regulated by HIF‐1α or other stress‐responsive transcription factors, and whether the *GSLR*‐DHX9‐CKB axis contributes to cellular adaptation to diverse metabolic stresses.

Furthermore, our preliminary TCGA pan‐cancer analysis revealed that *GSLR* is significantly upregulated in multiple cancer types, and high *GSLR* expression correlates with poor prognosis in several of these malignancies (Figure S10A, B). Consistent with this, aberrant upregulation of CKB has been reported to drive tumor progression in hepatocellular carcinoma [32], pancreatic adenocarcinoma [33], and glioblastoma [54]. These findings raise the possibility that the GSLR‐DHX9‐CKB axis may have broader relevance beyond LUAD. Further functional studies in additional tumor models will be needed to determine whether this pathway represents a shared metabolic adaptation mechanism and a potential therapeutic vulnerability across cancer types.

Several questions remain for future investigations. First, while our current study demonstrates that depletion of key metabolic cofactors and dysregulated expression of epigenetic enzymes collectively mediate histone modification remodeling at the GSLR locus under nutrient deprivation, the upstream nutrient sensors that link glucose/glutamine scarcity to these epigenetic effects remain to be identified. Second, although our findings support an indirect regulatory mode via metabolic cofactor intermediates, it remains to be systematically investigated whether glucose and glutamine also act as direct signaling cues to modulate lncRNAs transcription independently of metabolic flux. Finally, it will be important to determine whether the *GSLR–*DHX9–CKB axis is active in other oncogenic contexts and whether its inhibition synergizes with existing metabolic therapies.

In summary, we identified *GSLR* as a glucose/glutamine‐sensitive lncRNA that, via CTCF‐dependent transcription and interaction with DHX9, upregulates CKB to preserve mitochondrial function and restrain apoptosis in LUAD cells. Disrupting this *GSLR*‐DHX9‐CKB axis impairs energy balance and promotes cell death, revealing a metabolically regulated lncRNA pathway that may offer new opportunities for targeted therapy in LUAD (Figure 8I).

## Author Contributions

X.Q. designed and carried out the most experiments, performed statistical analysis, and generated the figures. J.Y. performed most animal assay and provided technical assistance. J.R. performed the bioinformatics analysis. X.M., L.D. and E.C. collected the tissue specimens and clinical data. J.L. conducted preparations for the RNA sequencing libraries. T.S. helped with the experiments. Y.L. and P.L. conceived the study. X.Q. wrote the initial manuscript. Y.L. and P.L. revised the manuscript. E.C., Y.L. and P.L. supervised the study. Y.L. and P.L. designed and oversaw the study.

## Conflicts of Interest

The authors declare no conflicts of interest.

## Supporting information




**Supporting File**: advs77950‐sup‐0001‐SuppMat.docx.

## Data Availability

The ChIP‐seq data analyzed in this study (H3K27ac, H3K9me3, and CTCF) were obtained from the Gene Expression Omnibus (GEO) database at accession numbers GSE91337, GSE91335, and GSM822289. The DRIP‐seq data analyzed in this study were obtained from the GEO at GSE300469. The raw RNA‐seq data generated in this study have been deposited in the Genome Sequence Archive (GSA) database under accession number HRA019339. All other raw data generated in this study are available from the corresponding author upon request.

## References

[1] F. Bray , M. Laversanne , H. Sung , et al., “Global Cancer Statistics 2022: Globocan Estimates of Incidence and Mortality Worldwide for 36 Cancers in 185 Countries,” CA: a cancer journal for clinicians 74, no. 3 (2024): 229–263, 10.3322/caac.21834. 38572751

[2] B. Han , R. Zheng , H. Zeng , et al., “Cancer Incidence and Mortality in China, 2022,” Journal of the National Cancer Center 4, no. 1 (2024): 47–53, 10.1016/j.jncc.2024.01.006. PMC11256708 39036382

[3] F. R. Hirsch , G. V. Scagliotti , J. L. Mulshine , et al., “Lung Cancer: Current Therapies and New Targeted Treatments,” The Lancet 389, no. 10066 (2017): 299–311, 10.1016/s0140-6736(16)30958-8. 27574741

[4] S. Rosner , J. E. Reuss , M. Zahurak , et al., “Five‐Year Clinical Outcomes after Neoadjuvant Nivolumab in Resectable Non–Small Cell Lung Cancer,” Clinical Cancer Research 29, no. 4 (2023): 705–710, 10.1158/1078-0432.Ccr-22-2994. PMC9932577 36794455

[5] D. Hanahan , “Hallmarks of Cancer: New Dimensions,” Cancer discovery 12, no. 1 (2022): 31–46, 10.1158/2159-8290.Cd-21-1059. 35022204

[6] G. Wang , M. Qiu , X. Xing , et al., “Lung Cancer Scrna‐Seq and Lipidomics Reveal Aberrant Lipid Metabolism for Early‐Stage Diagnosis,” Science translational medicine 14, no. 630 (2022): abk2756, 10.1126/scitranslmed.abk2756. 35108060

[7] J. Kim , H. M. Lee , F. Cai , et al., “The Hexosamine Biosynthesis Pathway Is a Targetable Liability in Kras/Lkb1 Mutant Lung Cancer,” Nature metabolism 2, no. 12 (2020): 1401–1412, 10.1038/s42255-020-00316-0. PMC7744327 33257855

[8] X. Wang , R. Liu , W. Zhu , et al., “Udp‐Glucose Accelerates Snai1 Mrna Decay and Impairs Lung Cancer Metastasis,” Nature 571, no. 7763 (2019): 127–131, 10.1038/s41586-019-1340-y. 31243371

[9] Y. Mao , Z. Xia , W. Xia , and P. Jiang , “Metabolic Reprogramming, Sensing, and Cancer Therapy,” Cell reports 43, no. 12 (2024): 115064, 10.1016/j.celrep.2024.115064. 39671294

[10] N. Kanarek , B. Petrova , and D. M. Sabatini , “Dietary Modifications for Enhanced Cancer Therapy,” Nature 579, no. 7800 (2020): 507–517, 10.1038/s41586-020-2124-0. 32214253

[11] Y. Xiao , T.‐J. Yu , Y. Xu , et al., “Emerging Therapies in Cancer Metabolism,” Cell metabolism 35, no. 8 (2023): 1283–1303, 10.1016/j.cmet.2023.07.006. 37557070

[12] Y.‐Q. Wu , C.‐S. Zhang , J. Xiong , et al., “Low Glucose Metabolite 3‐Phosphoglycerate Switches Phgdh from Serine Synthesis to P53 Activation to Control Cell Fate,” Cell research 33, no. 11 (2023): 835–850, 10.1038/s41422-023-00874-4. PMC10624847 37726403

[13] X. Liu , K. Olszewski , Y. Zhang , et al., “Cystine Transporter Regulation of Pentose Phosphate Pathway Dependency and Disulfide Stress Exposes a Targetable Metabolic Vulnerability in Cancer,” Nature cell biology 22, no. 4 (2020): 476–486, 10.1038/s41556-020-0496-x. PMC7194135 32231310

[14] R. W. Yao , Y. Wang , and L. L. Chen , “Cellular Functions of Long Noncoding Rnas,” Nature cell biology 21, no. 5 (2019): 542–551, 10.1038/s41556-019-0311-8. 31048766

[15] F. Kopp and J. T. Mendell , “Functional Classification and Experimental Dissection of Long Noncoding Rnas,” Cell 172, no. 3 (2018): 393–407, 10.1016/j.cell.2018.01.011. PMC5978744 29373828

[16] A. B. Herman , D. Tsitsipatis , and M. Gorospe , “Integrated Lncrna Function Upon Genomic and Epigenomic Regulation,” Molecular cell 82, no. 12 (2022): 2252–2266, 10.1016/j.molcel.2022.05.027. PMC9219586 35714586

[17] Y.‐T. Tan , J.‐F. Lin , T. Li , J.‐J. Li , R.‐H. Xu , and H.‐Q. Ju , “LncRNA‐Mediated Posttranslational Modifications and Reprogramming of Energy Metabolism in Cancer,” Cancer communications 41, no. 2 (2021): 109–120, 10.1002/cac2.12108. PMC7896749 33119215

[18] Y. Xu , M. Qiu , M. Shen , et al., “The Emerging Regulatory Roles of Long Non‐Coding Rnas Implicated in Cancer Metabolism,” Molecular Therapy 29, no. 7 (2021): 2209–2218, 10.1016/j.ymthe.2021.03.017. PMC8261164 33775912

[19] W. Lin , Q. Zhou , C.‐Q. Wang , et al., “Lncrnas Regulate Metabolism in Cancer,” International journal of biological sciences 16, no. 7 (2020): 1194–1206, 10.7150/ijbs.40769. PMC7053319 32174794

[20] R. C. Shankaraiah , A. Veronese , S. Sabbioni , and M. Negrini , “Non‐Coding Rnas in the Reprogramming of Glucose Metabolism in Cancer,” Cancer letters 419 (2018): 167–174, 10.1016/j.canlet.2018.01.048. 29366802

[21] W. Zhu , X. Chen , X. Guo , et al., “Low Glucose–Induced Overexpression of HOXC‐AS3 Promotes Metabolic Reprogramming of Breast Cancer,” Cancer research 82, no. 5 (2022): 805–818, 10.1158/0008-5472.Can-21-1179. 35031573

[22] Z. Lin , J. Song , Y. Gao , et al., “Hypoxia‐Induced Hif‐1α/Lncrna‐Pman Inhibits Ferroptosis by Promoting the Cytoplasmic Translocation of Elavl1 in Peritoneal Dissemination from Gastric Cancer,” Redox biology 52 (2022): 102312, 10.1016/j.redox.2022.102312. PMC9043498 35447413

[23] L. Sang , H.‐Q. Ju , Z. Yang , et al., “Mitochondrial Long Non‐Coding Rna Gas5 Tunes Tca Metabolism in Response to Nutrient Stress,” Nature metabolism 3, no. 1 (2021): 90–106, 10.1038/s42255-020-00325-z. 33398195

[24] X. Huang , L. Pan , Z. Zuo , et al., “Linc00842 Inactivates Transcription Co‐Regulator Pgc‐1α to Promote Pancreatic Cancer Malignancy through Metabolic Remodelling,” Nature communications 12, no. 1 (2021): 3830, 10.1038/s41467-021-23904-4. PMC8219694 34158490

[25] L. A. Sanz and F. Chédin , “High‐resolution, Strand‐specific R‐loop Mapping via S9.6‐based DNA–RNA Immunoprecipitation and High‐throughput Sequencing,” Nature protocols 14, no. 6 (2019): 1734–1755, 10.1038/s41596-019-0159-1. PMC6615061 31053798

[26] L. Sun , H. Zhang , and P. Gao , “Metabolic Reprogramming and Epigenetic Modifications on the Path to Cancer,” Protein & cell 13, no. 12 (2022): 877–919, 10.1007/s13238-021-00846-7. PMC9243210 34050894

[27] Y. C. Ng , W.‐C. Chung , H.‐R. Kang , et al., “A DNA‐sensing–independent Role of a Nuclear RNA Helicase, DHX9, in Stimulation of NF‐κB–mediated Innate Immunity against DNA Virus Infection,” Nucleic acids research 46, no. 17 (2018): 9011–9026, 10.1093/nar/gky742. PMC6158622 30137501

[28] M.‐Y. Liu , K.‐R. Lin , Y.‐L. Chien , et al., “Atr Phosphorylates Dhx9 at Serine 321 to Suppress R‐Loop Accumulation Upon Genotoxic Stress,” Nucleic acids research 52, no. 1 (2024): 204–222, 10.1093/nar/gkad973. PMC10783509 37930853

[29] B.‐Z. Yang , M.‐Y. Liu , K.‐L. Chiu , et al., “Dhx9 Sumoylation Is Required for the Suppression of R‐Loop‐Associated Genome Instability,” Nature communications 15, no. 1 (2024): 6009, 10.1038/s41467-024-50428-4. PMC11255299 39019926

[30] V. Frezza , L. Chellini , V. Riccioni , D. Bonvissuto , R. Palombo , and M. P. Paronetto , “Dhx9 Helicase Impacts on Splicing Decisions by Modulating U2 Snrnp Recruitment in Ewing Sarcoma Cells,” Nucleic Acids Research 53, no. 4 (2025), 10.1093/nar/gkaf068. PMC11826090 39950342

[31] J. Kim and J. Lee , “The Many Faces of the Creatine/Phosphocreatine System,” Nature metabolism 4, no. 2 (2022): 155–156, 10.1038/s42255-022-00539-3. 35169326

[32] K. Wu , M. Yan , T. Liu , et al., “Creatine Kinase B Suppresses Ferroptosis by Phosphorylating Gpx4 through a Moonlighting Function,” Nature cell biology 25, no. 5 (2023): 714–725, 10.1038/s41556-023-01133-9. 37156912

[33] V. Papalazarou , T. Zhang , N. R. Paul , et al., “The Creatine–phosphagen System is Mechanoresponsive in Pancreatic Adenocarcinoma and Fuels Invasion and Metastasis,” Nature metabolism 2, no. 1 (2020): 62–80, 10.1038/s42255-019-0159-z. PMC7617069 32694686

[34] T. Murayama , J. Nakayama , X. Jiang , et al., “Targeting Dhx9 Triggers Tumor‐Intrinsic Interferon Response and Replication Stress in Small Cell Lung Cancer,” Cancer discovery 14, no. 3 (2024): 468–491, 10.1158/2159-8290.Cd-23-0486. PMC10905673 38189443

[35] X. Ren , Q. Liu , P. Zhou , et al., “Dhx9 Maintains Epithelial Homeostasis by Restraining R‐Loop‐Mediated Genomic Instability in Intestinal Stem Cells,” Nature communications 15, no. 1 (2024): 3080, 10.1038/s41467-024-47235-2. PMC11004185 38594251

[36] J. F. Rahbani , A. Roesler , M. F. Hussain , et al., “Creatine Kinase B Controls Futile Creatine Cycling in Thermogenic Fat,” Nature 590, no. 7846 (2021): 480–485, 10.1038/s41586-021-03221-y. PMC8647628 33597756

[37] L. He , J. Lin , and S. Lu , “Ckb Promotes Mitochondrial Atp Production by Suppressing Permeability Transition Pore,” (2024), 2403093, 10.1002/advs.202403093. PMC11336976 38896801

[38] E. Vringer and S. W. G. Tait , “Mitochondria and Cell Death‐Associated Inflammation,” Cell Death & Differentiation 30, no. 2 (2023): 304–312, 10.1038/s41418-022-01094-w. PMC9950460 36447047

[39] I. Martínez‐Reyes and N. S. Chandel , “Cancer Metabolism: Looking Forward,” Nature reviews Cancer 21, no. 10 (2021): 669–680, 10.1038/s41568-021-00378-6. 34272515

[40] N. Hay , “Reprogramming Glucose Metabolism in Cancer: Can It Be Exploited for Cancer Therapy?,” Nature reviews Cancer 16, no. 10 (2016): 635–649, 10.1038/nrc.2016.77. PMC5516800 27634447

[41] L. Yang , S. Venneti , and D. Nagrath , “Glutaminolysis: A Hallmark of Cancer Metabolism,” Annual review of biomedical engineering 19, no. 1 (2017): 163–194, 10.1146/annurev-bioeng-071516-044546. 28301735

[42] M. Li , R. F. Thorne , and R. Shi , “Ddit3 Directs a Dual Mechanism to Balance Glycolysis and Oxidative Phosphorylation during Glutamine Deprivation,” (2021), 2003732, 10.1002/advs.202003732. PMC8188220 34105294

[43] G. L. Rampioni Vinciguerra , M. Capece , L. Reggiani Bonetti , et al., “Nutrient Restriction‐Activated Fra‐2 Promotes Tumor Progression via Igf1r in Mir‐15a Downmodulated Pancreatic Ductal Adenocarcinoma,” Signal transduction and targeted therapy 9, no. 1 (2024): 31, 10.1038/s41392-024-01740-4. PMC10859382 38342897

[44] L. Statello , C. J. Guo , L. L. Chen , and M. Huarte , “Gene Regulation by Long Non‐Coding Rnas and Its Biological Functions,” Nature reviews Molecular cell biology 22, no. 2 (2021): 96–118, 10.1038/s41580-020-00315-9. PMC7754182 33353982

[45] Y. Liu , X. Liu , C. Lin , et al., “Noncoding Rnas Regulate Alternative Splicing in Cancer,” Journal of Experimental & Clinical Cancer Research 40, no. 1 (2021): 11, 10.1186/s13046-020-01798-2. PMC7789004 33407694

[46] H. Yang , Y. Hu , M. Weng , et al., “Hypoxia Inducible Lncrna‐Cbslr Modulates Ferroptosis through M6a‐Ythdf2‐Dependent Modulation of Cbs in Gastric Cancer,” Journal of advanced research 37 (2022): 91–106, 10.1016/j.jare.2021.10.001. PMC9039740 35499052

[47] X. Ren , D. Wang , G. Zhang , et al., “Nucleic Dhx9 Cooperates with Stat1 to Transcribe Interferon‐Stimulated Genes,” Science advances 9, no. 5 (2023): add5005, 10.1126/sciadv.add5005. PMC9897671 36735791

[48] M. M. Suzuki , K. Iijima , K. Ogami , et al., “Tug1‐Mediated R‐Loop Resolution at Microsatellite Loci as a Prerequisite for Cancer Cell Proliferation,” Nature communications 14, no. 1 (2023): 4521, 10.1038/s41467-023-40243-8. PMC10444773 37607907

[49] P. Hou , S. Meng , M. Li , et al., “Linc00460/Dhx9/Igf2bp2 Complex Promotes Colorectal Cancer Proliferation and Metastasis by Mediating Hmga1 Mrna Stability Depending on M6a Modification,” Journal of Experimental & Clinical Cancer Research 40, no. 1 (2021): 52, 10.1186/s13046-021-01857-2. PMC7851923 33526059

[50] Y. Sun , H. Zhang , R. Ma , et al., “Ets‐1‐Activated Linc01016 over‐Expression Promotes Tumor Progression via Suppression of Rffl‐Mediated Dhx9 Ubiquitination Degradation in Breast Cancers,” Cell death & disease 14, no. 8 (2023): 507, 10.1038/s41419-023-06016-3. PMC10406855 37550275

[51] K. Yang , W. Zhang , L. Zhong , et al., “Long Non‐Coding Rna Hif1a‐As2 and Myc Form a Double‐Positive Feedback Loop to Promote Cell Proliferation and Metastasis in Kras‐Driven Non‐Small Cell Lung Cancer,” Cell Death & Differentiation 30, no. 6 (2023): 1533–1549, 10.1038/s41418-023-01160-x. PMC10089381 37041291

[52] Y. Lou , W.‐H. Tian , J.‐L. Cui , et al., “Miat‐Dhx9 Spatiotemporal Expression Drives Venous Neointimal Hyperplasia through Nucleolar Homeostasis and Mitotic Progression,” Cell reports 44, no. 12 (2025): 116578, 10.1016/j.celrep.2025.116578. 41259202

[53] L. Zhang and P. Bu , “The Two Sides of Creatine in Cancer,” Trends in cell biology 32, no. 5 (2022): 380–390, 10.1016/j.tcb.2021.11.004. 34895811

[54] L. Chen , Q. Qi , X. Jiang , et al., “Phosphocreatine Promotes Epigenetic Reprogramming to Facilitate Glioblastoma Growth through Stabilizing Brd2,” Cancer discovery 14, no. 8 (2024): 1547–1565, 10.1158/2159-8290.Cd-23-1348. 38563585

[55] M. Tlili , B. Samborska , C. Girondel , et al., “Creatine Kinase B Promotes Non–small Cell Lung Cancer Survival and Metastasis,” Journal of Biological Chemistry 301, no. 11 (2025): 110805, 10.1016/j.jbc.2025.110805. PMC12630352 41072771

[56] J. Duan , Z. Huang , E. C. Nice , N. Xie , M. Chen , and C. Huang , “Current Advancements and Future Perspectives of Long Noncoding Rnas in Lipid Metabolism and Signaling,” Journal of advanced research 48 (2023): 105–123, 10.1016/j.jare.2022.08.007. PMC10248733 35973552

[57] R. Liu , Y. Guo , L. Wang , et al., “A Novel Hypoxia‐Induced Lncrna, Szt2‐As1, Boosts Hcc Progression by Mediating Hif Heterodimerization and Histone Trimethylation under a Hypoxic Microenvironment,” Cell Death & Differentiation 32, no. 4 (2025): 714–729, 10.1038/s41418-024-01419-x. PMC11982551 39572656

[58] Z. Chen , C. Chen , L. Xiao , et al., “Hilps, a Long Noncoding Rna Essential for Global Oxygen Sensing in Humans,” Science advances 9, no. 47 (2023): adi1867, 10.1126/sciadv.adi1867. PMC10664984 37992175

