# Supplementary material for: GSLR Drives Lung Adenocarcinoma as a Critical Regulator of Energy Homeostasis Through Creatine Kinase B Activation
Source: Adv Sci (Weinh). 2026 Sep 27:e77950. Online ahead of print. doi: 10.1002/advs.77950 (PMC13616385; doi:10.1002/advs.77950)
Supplement: Supplementary file 1 [file ADVS-9999-e77950-s001.docx]

**Table S1. Sequences of primers and siRNAs used in this study.**

| Oligo Name | | Sequence (5'-3') |
| --- | --- | --- |
| *GSLR* qPCR primer | Forward | GGGAAGACCAGAGGCATCAC |
|  | Reverse | GCCTCCTGTCAATGGAACCT |
| DHX9 qPCR primer | Forward | TCCAACTGGAATCCTTGGAC |
|  | Reverse | TTTTCCCACATCCAGTAGCC |
| CKB qPCR primer | Forward | CCGTACATCATGACCGTGGG |
|  | Reverse | CCCTGCAGGTTGTCGGG |
| ACTB qPCR primer | Forward | GGACTTCGAGCAAGAGATGG |
|  | Reverse | AGCACTGTGTTGGCGTACAG |
| GAPDH qPCR primer | Forward | AGCTCACTGGCATGGCCTTC |
|  | Reverse | CGCCTGCTTCACCACCTTCT |
| TCF21 qPCR primer | Forward | CACTTGAGGCAGATCCTGGCTA |
|  | Reverse | CGGTCACCACTTCTTTCAGGTC |
| NEAT1 primer | Forward | TTTGTGCTTGGAACCTTGCT |
|  | Reverse | TCAACGCCCCAAGTTATTTC |
| U1 snRNA primer | Forward | GGGAGATACCATGATCACGAAGGT |
|  | Reverse | CCACAAATTATGCAGTCGAGTTTCCC |
| *GSLR* ChIP primer | Forward | CCTACTCAAGATGGAGTCGCT |
|  | Reverse | AGATGATTTGTAGACCCTGCAC |
| CKB ChIP primer | Forward | AGGATGCGGATGAACCCAAG |
|  | Reverse | GGATATCTGGATCCCCTCGC |
| *GSLR*-T7 FL | Forward | TAATACGACTCACTATAGGGCGTGGGATCCGCCCCTCC |
|  | Reverse | TTTTTAAGTGACCATTTCTTAGTTGACA |
| *GSLR*-T7 Frag.1 | Forward | TAATACGACTCACTATAGGGCGTGGGATCCGCCCCTCC |
|  | Reverse | GGCAGGGGAGGCTCGGC |
| *GSLR*-T7 Frag.2 | Forward | TAATACGACTCACTATAGGGTCCTGGCCGCCTCTGCA |
|  | Reverse | TGACCTGCACACAGACAGTG |
| *GSLR*-T7 Frag.3 | Forward | TAATACGACTCACTATAGGGGGCAGGGGATGCTGGTG |
|  | Reverse | AGCCATGACTCCACCACCA |
| *GSLR*-T7 Frag.4 | Forward | TAATACGACTCACTATAGGGGCAGGCCAGGGAGATGC |
|  | Reverse | TTTTTAAGTGACCATTTCTTAGTTGACA |
| pGL3-*GSLR* promoter | Forward | gcgtgctagcccgggctcgagATGAGCTGCTCGAGGGAGTATACT |
|  | Reverse | cagtaccggaatgccaagcttAGGGCAGGCGCGCCCAGG |

**Table S1 (continued). Sequences of primers and siRNAs used in this study.**

| Oligo Name | | Sequence (5'-3') |
| --- | --- | --- |
| pcDHA-DHX9-FLAG MUT1 | Forward | cttggtaccgagctcgaattcATGGGTGACGTTAAAAATTTTCTGT |
|  | Reverse | atggtctttgtagtcggatccCGGGGGCAAAATCTCAAGA |
| pcDHA-DHX9-FLAG MUT2 | Forward | cttggtaccgagctcgaattcATGCCTGAAGATCCTTCTGTGCCA |
|  | Reverse | atggtctttgtagtcggatccAAAACGTATCTCTGCTTCTTCTCCA |
| pcDHA-DHX9-FLAG MUT3 | Forward | cttggtaccgagctcgaattcATGGGTGACGTTAAAAATTTTCTGT |
|  | Reverse | atggtctttgtagtcggatccAAAACGTATCTCTGCTTCTTCTCCA |
| pcDHA-DHX9-FLAG MUT4 | Forward | cttggtaccgagctcgaattcATGCCTGAAGATCCTTCTGTGCCA |
|  | Reverse | atggtctttgtagtcggatccATAGCCGCCACCTCCTCTTC |
| pGL3-CKB promoter | Forward | gcgtgctagcccgggctcgagGGCCTTCTGAGAAGCTGCAG |
|  | Reverse | cagtaccggaatgccaagcttTTGTGGCTGTTGGAGAAGGG |
| *GSLR* siRNA2 | Sense | UUUCCCUGAGGUCUCUCUUCCTT |
|  | Antisense | GGAAGAGAGACCUCAGGGAAATT |
| *GSLR* siRNA3 | Sense | UUCAUGUACACCCAAGACACCTT |
|  | Antisense | GGUGUCUUGGGUGUACAUGAATT |
| DHX9 siRNA1 | Sense | UAUAUCCGCUUCCAUUGUCGUTT |
|  | Antisense | ACGACAAUGGAAGCGGAUAUATT |
| DHX9 siRNA2 | Sense | UUUCUUGAGUAGUAAUCCUUCTT |
|  | Antisense | GAAGGAUUACUACUCAAGAAATT |
| CKB siRNA1 | Sense | AUGAACUCAUAGUCCUUAGACTT |
|  | Antisense | GUCUAAGGACUAUGAGUUCAUTT |
| CKB siRNA2 | Sense | UCAUGCUCUUGAGCGCGUAGUTT |
|  | Antisense | ACUACGCGCUCAAGAGCAUGATT |
| NFKB1 siRNA | Sense | CCAGAGUUUACAUCUGAUGAUTT |
|  | Antisense | AUCAUCAGAUGUAAACUCUGGTT |
| RelA siRNA | Sense | CACCAUCAACUAUGAUGAGUUTT |
|  | Antisense | AACUCAUCAUAGUUGAUGGUGTT |
| SP1 siRNA | Sense | GCUGGUGGUGAUGGAAUACAUTT |
|  | Antisense | AUGUAUUCCAUCACCACCAGCTT |
| MAZ siRNA | Sense | GAUGCUGAGCUCGGCUUAUAUTT |
|  | Antisense | AUAUAAGCCGAGCUCAGCAUCTT |
| CTCF siRNA | Sense | CAAGAAUGAGAAGCGCUUUAATT |
|  | Antisense | UUAAAGCGCUUCUCAUUCUUGTT |

**Table S2. Information of antibodies used in this study.**

| **Antibody** | **Supplier** | **Catalogue** | **Species** | **Applications** |
| --- | --- | --- | --- | --- |
| DHX9 | Proteintech | 17721-1-AP | Rabbit | WB, RIP, ChIP, IF |
| CKB | Santa Cruz | sc-373686 | Mouse | WB, IHC |
| Bcl-2 | Proteintech | 12789-1-AP | Rabbit | WB |
| Bax | Proteintech | 50599-2-Ig | Rabbit | WB |
| CTCF | Cell Signaling Technology | #3418 | Rabbit | WB, ChIP |
| PARP | Cell Signaling Technology | #9542 | Rabbit | WB |
| Caspase 7 | Cell Signaling Technology | #9492 | Rabbit | WB |
| Caspase 3 | Cell Signaling Technology | # 9662 | Rabbit | WB |
| Cleaved Caspase-3 | Cell Signaling Technology | #9664 | Rabbit | IHC |
| H3K4me3 | Cell Signaling Technology | #9751 | Rabbit | WB, ChIP |
| H3K9me3 | Cell Signaling Technology | #13969 | Rabbit | WB, ChIP |
| H3K27me3 | Cell Signaling Technology | #9733 | Rabbit | WB, ChIP |
| H3K27ac | Cell Signaling Technology | #8173 | Rabbit | WB, ChIP |
| S9.6 | Millipore | MABE1095 | Mouse | DRIP |
| GAPDH | Proteintech | 60004-1-Ig | Mouse | WB |
| β-actin | Proteintech | 66009-1-Ig | Mouse | WB |
| HRP-conjugated Goat Anti-Rabbit IgG | Cell Signaling Technology | #7074 | Goat | WB |
| HRP-conjugated Goat Anti-Mouse IgG | Cell Signaling Technology | #7076 | Goat | WB |

**Figure S1. Identification of *GSLR* as a metabolism-related lncRNA**

**(A)** Schematic diagram showing the genomic location of GSLR at human chromosome 15q13.1.

**(B)** qPCR assays were performed to quantify the expression of *GSLR* at different points of glutamine deprivation (0-24h).

**(C)** qPCR assays were performed to quantify the expression of *GSLR* after treated with different concentrations of glutamine (0-1mM) for 24h.

**(D)** qPCR assays were performed to quantify the recovery of *GSLR* expression at different time points (0-12h) after glutamine deprivation (24h) followed by restoration to normal culture conditions.

**(E, F)** The nuclear-cytoplasmic fractionation assays were used to detect the subcellular localization of *GSLR*; GADPH and ACTB served as cytoplasmic controls, while NEAT1 and U1 snRNA served as nuclear controls.

**(G)** Fluorescence in situ hybridization assays were used to exhibit the subcellular localization of *GSLR*; U6 served as a nuclear control, and 18S served as a cytoplasmic control; scale bar, 10 µm.

**(H)** The protein coding potential of *GSLR* evaluated by Coding Potential Calculator 2 (CPC2). ACTB and GAPDH served as coding gene controls, while XIST and HOTAIR served as non-coding controls for the prediction.

Data are represented as mean±S.D. Statistical significance was determined by one-way ANOVA followed by Tukey's post-hoc test (A-C), ***p < 0.001, ns = not significant.

**Figure S2. Histone modifications and CTCF regulate the transcription of *GSLR*.**

**(A, B)** qPCR analysis of *GSLR* levels after treatment with different doses of 5-Aza-2ʹ-Deoxycytidine (5-Aza-dC) for 48 (A) or 72 (B) hours. TCF21 served as the positive control.

**(C)** Western blot assays were performed to detect the histone modifications (H3K4me3, H3K9me3, H3K27me3, and H3K27ac) changes under glucose or glutamine deprivation. Numbers below each lane represent the relative quantification of band intensity normalized to ACTB.

**(D)** Western blot analysis of CBP, HDAC1, EZH2 and SIRT1 protein levels in A549 and H1650 cells subjected to glucose deprivation (Glu (−)) or glutamine deprivation (Gln (−)) for the indicated time points (0, 12, 24 h).

**(E, F)** Quantification of intracellular α-ketoglutarate (α-KG) (E) and acetyl-CoA (F) levels in A549 cells under normal culture (Ctr) and glucose deprivation (Glu (−)) conditions.

**(G)** qPCR assays quantifying the effect of candidate transcription factors on *GSLR* expression in H1650 cells.

**(H)** ChIP-seq track (GSM822289) showing CTCF enrichment at the *GSLR* promoter.

Data are represented as mean±S.D. Statistical significance was determined by one-way ANOVA followed by Tukey's post-hoc test, *p < 0.05, **p < 0.01, ***p < 0.001, ns = not significant.

**Figure S3. *GSLR* promotes cell proliferation in LUAD.**

**(A)** qPCR analysis of *GSLR* expression in A549 and H1650 cells transfected with sh-Ctrl and sh-*GSLR*.

**(B, C)** CCK-8 assays assessing cell proliferation in sh-Ctrl and sh-*GSLR* cells.

**(D)** Colony formation assays assessing colony formation abilities in sh-Ctrl and sh-*GSLR* cells.

**(E)** qPCR analysis of *GSLR* expression in A549 and H1650 cells transfected with pcDNA3.1 and *GSLR*-OE plasmid.

**(F, G)** CCK-8 assays assessing cell proliferation in pcDNA3.1 and *GSLR*-OE cells.

**(H)** Colony formation assays assessing colony formation abilities in pcDNA3.1 and *GSLR*-OE cells.

Data are represented as mean±S.D. Statistical significance was determined by two-tailed unpaired Student's t-test, **p < 0.01, ***p < 0.001.

**Figure S4. *GSLR* directly associates with DHX9.**

**(A)** The LC-MS/MS mass spectrometry detected the specific peptide segment of DHX9.

**(B)** Western blot analyzed the immunoprecipitation efficiency of DHX9 in the RIP assays (Related to Fig.4E).

**(C)** The schematic diagram of *GSLR* secondary structure predicted by RNAfold software. *GSLR* was truncated into four fragments based on its secondary structure.

**(D)** Western blot analyzed the immunoprecipitation efficiency of FLAG antibody for the ectopically expressed full-length and mutant forms of DHX9 (Related to Fig.4L).

**(E)** Representative agarose gel electrophoresis image showing the immunoprecipitation efficiency of the full-length and mutant forms of DHX9 for *GSLR*.

**Figure S5. DHX9 promotes cell proliferation and metastasis of LUAD.**

**(A)** Western blot quantitative analysis of DHX9 protein expression in paired lung adenocarcinoma tissues and adjacent normal tissues, n = 7, N: normal, T: tumor.

**(B)** qPCR assays were performed to determine the interference efficiency of shRNA on the level of DHX9 mRNA.

**(C, D)** CCK-8 assays were performed to assess cell proliferation in shCtrl and shDHX9 lung cancer cells.

**(E)** Colony formation assays were performed to assess colony formation abilities in shCtrl and shDHX9 lung cancer cells.

**(F, G)** CCK-8 assays assessing cell proliferation in A549 (F) and H1650 (G) cells transfected with empty vector (pcDNA3.1), DHX9-FL, or DHX9 ΔOB-fold.

**(H)** Representative images of colony formation assays in A549 and H1650 cells transfected with the constructs described above.

**(I, J)** CCK-8 assays assessing cell proliferation in A549 (I) and H1650 (J) cells transfected with empty vector (pcDNA3.1), *GSLR*-FL, or *GSLR* ΔT4.

**(K)** Representative images of colony formation assays in A549 and H1650 cells transfected with the constructs described above.

Data are represented as mean±S.D. Statistical significance was determined by one-way ANOVA followed by Tukey's post-hoc test (B-D, F, G, I, J), *p < 0.05, **p < 0.01, ***p < 0.001, ns = not significant.

**Figure S6. CKB is a downstream target gene of the *GSLR*/DHX9 complex.**

**(A)** The volcano plot showing the protein-coding genes that are differentially expressed under glucose deprivation.

**(B)** The volcano plot showing the protein-coding genes that are differentially expressed under glutamine deprivation.

**(C)** The volcano plot showing the protein-coding genes that are differentially expressed after *GSLR* knockdown.

**(D)** The volcano plot showing the protein-coding genes that are differentially expressed after DHX9 knockdown.

**(E)** Venn diagram showing overlap of differentially expressed genes from four transcriptomic conditions: glucose deprivation, glutamine deprivation, *GSLR* knockdown, and DHX9 knockdown.

**(F)** Heatmap displaying expression profiles of 36 intersecting genes across the four conditions.

**(G, H)** qPCR and western blot analysis of CKB expression over time (0-24h) under glutamine deprivation.

**(I, J)** qPCR and western blot analysis of CKB expression after treated with varying glutamine concentrations (0-1mM) for 24h.

**(K)** qPCR analyzed the effects of individually knocking down *GSLR* or DHX9, as well as combined knockdown of *GSLR* and DHX9, on the expression level of *CKB* mRNA.

**(L)** Western blot assays detected the effects of individually knocking down *GSLR* or DHX9, as well as the combined knockdown of *GSLR* and DHX9, on the expression level of CKB protein.

(**M**) Quantitative analysis of CKB protein levels from western blot assays shown in (L). Band intensities were quantified using ImageJ and normalized to ACTB.

**(N)** qPCR analysis of CKB mRNA levels in A549 and H1650 cells transfected with pcDNA3.1, *GSLR* FL, or *GSLR* ΔT4.

**(O)** qPCR analysis of CKB mRNA levels in A549 and H1650 cells transfected with pcDNA3.1, DHX9 FL, or DHX9 ΔOB-fold.

Data are represented as mean±S.D. Statistical significance was determined by one-way ANOVA followed by Tukey's post-hoc test (G, I, K, M-O), *p < 0.05, **p < 0.01, ***p < 0.001, ns = not significant.

**Figure S7. CKB promotes cell proliferation in LUAD.**

**(A)** Western blot analysis of CKB expression in A549 and H1650 cells transfected with two independent siRNAs (siCKB-1, siCKB-2) versus negative control siRNA (siNC).

**(B, C)** CCK-8 assays assessing cell proliferation in siNC and siCKB cells.

**(D)** Colony formation assays assessing clonogenic potential in siNC and siCKB cells.

**(E, F)** EdU incorporation assays (E) and quantification (F) assessing DNA-replication rates in siNC and siCKB cells; scale bar, 100 µm.

**(G)** Western blot analysis of CKB expression in A549 and H1650 cells transfected with pcDNA3.1 or CKB-OE plasmid.

**(H, I)** CCK-8 assays assessing cell proliferation in pcDNA3.1 and CKB-OE cells.

**(J)** Colony formation assays assessing clonogenic potential in pcDNA3.1 and CKB-OE cells.

**(K)** Quantification of migrated cells in A549 and H1650 cells transfected with shCtrl+pcDNA3.1, shCtrl+CKB-OE, sh*GSLR*+pcDNA3.1, or sh*GSLR*+CKB-OE.

**(L, M)** ChIP-qPCR and agarose gel electrophoresis showing reduced DHX9 occupancy at the CKB promoter under glucose deprivation.

Data are represented as mean±S.D. Statistical significance was determined by one-way ANOVA followed by Tukey's post-hoc test (B, C, F, K) or two-tailed unpaired Student's t-test (H, I, L). *p < 0.05, **p < 0.01, ***p < 0.001, ns = not significant.

**Figure S8. *GSLR*/DHX9/CKB axis regulates mitochondrial-dependent apoptosis**

**(A)** Confocal microscopy images show the effects of *GSLR* and DHX9 knockdown on mitochondrial membrane potential; the A549 cells were treated with 4 μM JC-1 probe for 30 minutes; scale bar, 25 μm.

**(B)** Confocal microscopy images show the effect of glucose deprivation on mitochondrial membrane potential; the H1650 cells were treated with 4 μM JC-1 probe for 30 minutes; scale bar, 25 μm.

**(C)** Flow cytometry analysis of intracellular ROS levels under glucose deprivation. The H1650 cells were treated with a 10 μM DCFH-DA probe for 20 min.

**(D)** Quantification of DCF fluorescence intensity in (C) from three independent experiments.

**(E)** Flow cytometry analysis of intracellular ROS levels after knockdown of *GSLR*, DHX9 and CKB; the H1650 cells were treated with a 10 μM DCFH-DA probe for 20 min.

**(F)** Quantification of DCF fluorescence intensity in (E) from three independent experiments.

**(G)** Flow cytometry assays were performed to detect the cell apoptosis after *GSLR*, DHX9 and CKB knockdown.

Data are represented as mean±S.D. Statistical significance was determined by two-tailed unpaired Student's t-test (D) or one-way ANOVA followed by Tukey's post-hoc test (F), ***p < 0.001.

**Figure S9. *GSLR*‑DHX9 interaction is specific and non‑perturbing**

**(A)** Violin plots showing the expression levels of *GSLR* and other DHX9 binding lncRNAs in the TCGA‑LUAD cohort.

**(B)** RT‑qPCR quantification of relative expression of *GSLR* and other DHX9 binding RNAs in A549 cells.

**(C)** RIP-qPCR analysis of DHX9 enrichment efficiency for *GSLR* and other interacting RNAs.

**(D)** RIP-qPCR analysis of a panel of well‑documented DHX9‑binding RNAs in control (NC) and *GSLR*‑knockdown (*GSLR* KD) A549 cells.

**Figure S10. Pan-cancer expression and prognostic analysis of *GSLR*.**

**(A)** Box plots showing *GSLR* mRNA expression levels in tumor tissues (red) and paired adjacent normal tissues (blue) across 23 cancer types from the TCGA dataset.

**(B)** Heatmap showing the hazard ratios (HR) for overall survival associated with *GSLR* expression in different cancer types. Red indicates high *GSLR* expression correlates with poor prognosis, while blue indicates high *GSLR* expression correlates with favorable prognosis. The color intensity represents the log₁₀(HR) value. Bold boxes highlight statistically significant results (p < 0.05).
